# Coherent structures in active flows on dynamic surfaces

**DOI:** 10.1093/pnasnexus/pgag335

**Published:** 2026-09-30

**Authors:** Sreejith Santhosh, Cuncheng Zhu, Blase Fencil, Mattia Serra

**Affiliations:** Department of Physics, University of California, San Diego, CA 92093, USA; Department of Mechanical Engineering, University of California, San Diego, CA 92093, USA; Department of Physics, University of California, San Diego, CA 92093, USA; Department of Physics, University of California, San Diego, CA 92093, USA

**Keywords:** morphogenesis, coherent structures, tissue flows, active matter

## Abstract

Coherent structures—flow features that organize material transport and deformation—are central to analyzing complex flows in fluids, plasmas, and active matter. Yet, identifying such structures on dynamic surfaces remains an open challenge, limiting their application to many living and synthetic systems. Here, we introduce a geometric framework to extract Lagrangian and Eulerian coherent structures from velocity data on arbitrarily shaped, time-evolving surfaces. Our method operates directly on triangulated meshes, is parameterization-free, objective, and robust to noise. Applying this framework to active nematic vesicles, collectively migrating epithelial spheroids, and beating zebrafish hearts, we uncover transport barriers and Lagrangian deformation patterns—such as dynamic attractors, repellers, isotropic, and anisotropic strain—missed by conventional Eulerian analyses. This approach offers a new perspective on soft and living matter, revealing how geometry and activity can be harnessed to program synthetic materials, and how Lagrangian strain and principal deformation directions can help uncover mechanosensitive processes and directional cues in morphogenesis.

Significance statement Many biological processes—from embryonic development to collective cell migration—occur on curved, evolving surfaces. This work introduces a parameterization-free, geometric method to quantify transport and deformation patterns on dynamic surfaces of arbitrary geometry and topology directly from noisy velocity data. By identifying coherent structures on time-evolving surfaces defined by a mesh, our approach reveals attractors, repellers, and principal deformation directions in both synthetic active materials and living tissues. These frame-invariant quantities link surface geometry, activity, and deformation, opening new avenues for understanding morphogenesis mechanisms and for engineering active matter.

## Introduction

Active matter flows on curved surfaces are ubiquitous in biological systems, spanning morphogenesis (1–3), cytoskeletal remodeling (4, 5), in vitro epithelial spheroids (6–9), and synthetic active nematics (10, 11). Recent advances in live imaging and computation have enabled the reconstruction of such flows across systems ranging from developing embryos to engineered active nematics (12–16). These flows are spatially heterogeneous and temporally unsteady, driven by internal activity and influenced by surface curvature and topology (9, 11, 17). While kinematic velocity or trajectory data become increasingly available, its analysis remains challenging, yet essential for controlling material transport or discovering the underlying biophysical mechanisms.

Lagrangian and Eulerian coherent structures—denoted together as (CSs)—provide a reduced representation of complex trajectories, organizing material transport and deformation (18–21). CSs are typically objective (22)—i.e. invariant to the choice of coordinate system adopted to describe motion—and robust to noise, hence ideal for analyzing and comparing experiments. CSs have been broadly applied in science and engineering (23), including atmospheric and oceanic flows (24–27), turbulence (28, 29), plasmas (30), cardiovascular flows (31), bioinspired flows (32), as well as to study transport of plankton in jellyfish feeding (33) and dispersion of plant pathogens (34). In active nematics, recent works elucidated the connection between CSs and topological defect motion, revealing the interplay between flow structure and nematic order (35–37).

Lagrangian coherent structures (LCSs) have revealed cumulative deformation patterns and dynamic boundaries in multicellular flows—robust to noise and experimental variability—while remaining undetected by Eulerian quantities (38). These structures provided insights into morphogenetic mechanisms (39, 40), pattern formation (41), tissue mechanics (42), and cell fate bifurcations (43, 44). It has also provided a framework to engineer material transport in active systems (45). While existing CS techniques cover general 2D and 3D flows, flows defined by sparse, noisy trajectories (46–49), or flows on static surfaces (38, 50), they remain unavailable for flows on dynamic surfaces—ubiquitous in natural and synthetic active systems.

Here, we introduce a theoretical and computational framework to identify LCS and Eulerian CS in flows on arbitrary dynamic surfaces. Our method estimates deformation tensors from velocity data defined on triangulated meshes, is parameterization-free and extends the CS framework to evolving surfaces. We apply this framework to nematic flows on deforming vesicles, collective migration in pancreatic spheroids, and cardiac tissue deformations in embryonic zebrafish hearts—revealing undocumented dynamic attractors, repellers, and deformation patterns. These CSs and directional patterns of maximal stretch or contraction—absent in instantaneous velocity or nematic fields (Fig. 1)—can help uncover emergent mechanical properties and mechanotransduction mechanisms in living and synthetic active matter using only available kinematic data. We provide a brief tutorial (link) and MATLAB code (link) to facilitate use by the biological and soft matter communities.

**Figure 1:**
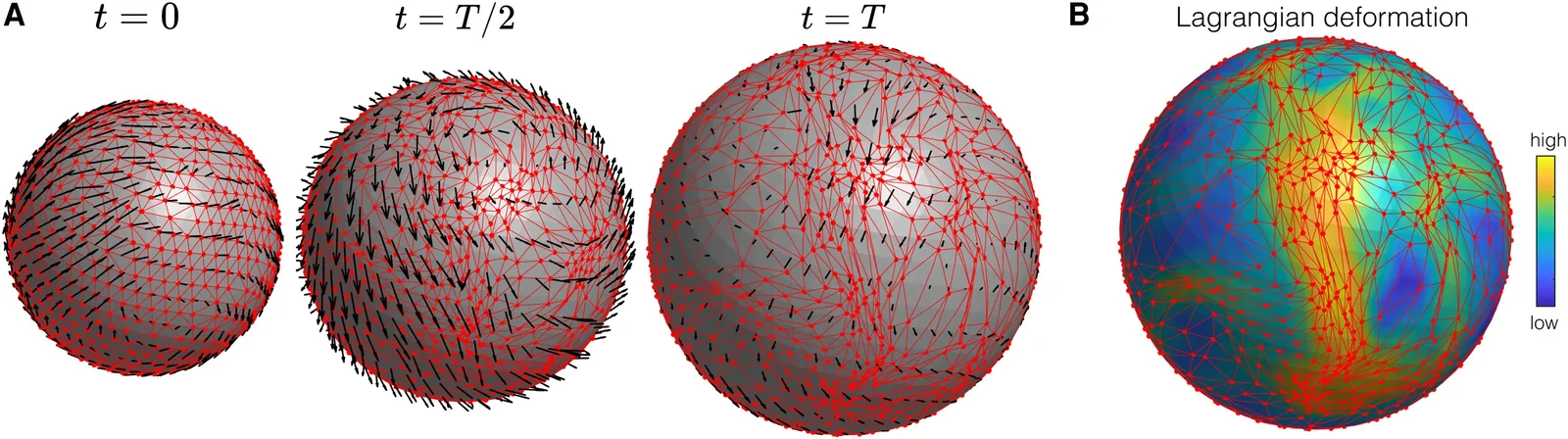
LCSs provide the geometric skeleton of complex flow fields. A) Snapshots of unsteady velocity fields (black arrows) on a growing spherical surface and the corresponding Lagrangian deformation grid (red grid). B) Backward FTLE field $\Lambda_{T}^{0}(x_{T})$ visualized on the final surface at t=T for trajectories over [0,T]. Large values (yellow) highlight regions of strong material attraction, corroborated by the deformed Lagrangian grid but not apparent in instantaneous Eulerian velocity snapshots.

## Results

### LCSs on dynamic surfaces

Flows on dynamic surfaces are represented by a 3D velocity field v(x,t) defined on a time-dependent 2D surface M(t). M(t) is typically provided in the form of a triangulated mesh (a set of connected points in 3D) on which v(x,t) is measured or simulated. Throughout, we denote vectors and matrices in bold and scalars in normal font. To study material transport and deformations of these flows over a time-interval [0,t], we define the flow (or trajectory) map


(1) $$F_{0}^{t}(x_{0})=x_{0}+\int_{0}^{t}v(F_{0}^{\tau }(x_{0}),\tau )d\tau ,$$

which maps the initial position $\left(x_{0},y_{0},z_{0})=x_{0}\in M(0\right)$ of a material particle to its final position $x_{t}=F_{0}^{t}(x_{0})\in M(t)$ (Fig. 2A, B). The deformation experienced by a material patch in the neighborhood of $x_{0}$ is encoded in


(2) $$(B_{0}^{t}(x_{0}){)}^{ij}=\zeta^{i}(x_{t},t{)}^{⊤}\nabla_{x_{0}}F_{0}^{t}(x_{0})\zeta^{j}(x_{0},0),$$

where $\left[\zeta^{1}(x,t),\zeta^{2}(x,t)\right]$ are orthonormal basis vectors for the tangent space $T_{x}M(t)$ (Fig. 2C), $\nabla_{x_{0}}F_{0}^{t}(x_{0})$ is the flow map gradient and ${\;}^{⊤}$ denotes vector transposition (Section S1 and Fig. S1 for derivation). Here, the superscripts i=1,2 on $\zeta^{i}$ label the two orthonormal tangent-space basis vectors; they do not denote contravariant tensor indices. To visualize $B_{0}^{t}(x_{0})$, consider $\eta_{0}\in T_{x_{0}}M(0)$—an infinitesimal relative position vector between neighboring trajectories, projected on the tangent space at $x_{0}$ using the basis vectors $\left[\zeta^{1}(x_{0},0),\zeta^{2}(x_{0},0)\right]$. $\eta_{0}$ moves and deforms to $\eta_{t}=B_{0}^{t}(x_{0})\eta_{0}\in T_{x_{t}}M(t)$, represented in the corresponding, orthonormal basis $\left[\zeta^{1}(x_{t},t),\zeta^{2}(x_{t},t)\right]$ (Fig. 2C, Section S1). The maximum (minimum) stretching experienced by any $\eta_{0}\in T_{x_{0}}M(0)$ at $x_{0}$ corresponds to the largest singular value $\lambda_{0}^{t}(x_{0})$ (smallest ${\;}_{m}\lambda_{0}^{t}(x_{0})$) of $B_{0}^{t}(x_{0})$, from which one can compute the largest finite-time Lyapunov exponent (FTLE)


(3) $$\Lambda_{0}^{t}(x_{0})=\text{log}(\lambda_{0}^{t}(x_{0}))/|t|.$$

The local $\eta_{0}$ at $x_{0}$ which undergoes maximal stretching corresponds to the largest singular vector $\xi_{0}^{t}(x_{0})$ of $B_{0}^{t}(x_{0})$. Isotropic Lagrangian deformation can be quantified by


(4) $${\;}_{\mathrm{iso}}\Lambda_{0}^{t}(x_{0})=\text{log}(|\text{det}(B_{0}^{t}(x_{0}))|)/|t|,$$

where $\left|\text{det}(B_{0}^{t}(x_{0}))\right|$ represents the ratio of the final area (time *t*) to the initial (time 0) area of an infinitesimal material patch starting at $x_{0}$ (Fig. 2B, C). Similarly, the anisotropy of Lagrangian deformation can be quantified by


(5) $${\;}_{\mathrm{aniso}}\Lambda_{0}^{t}(x_{0})=\text{log}(\lambda_{0}^{t}(x_{0})/{\;}_{m}\lambda_{0}^{t}(x_{0})).$$

Regions of high $\Lambda_{0}^{t}(x_{0})$—i.e. forward-time FTLE—mark initial domains where initially close particles will maximally separate during [0,t], locating *repelling LCS* (18, 19, 51), hereafter called *repellers*. Similarly, the backward FTLE $\Lambda_{t}^{0}(x_{t})$ field—computable by integrating trajectories in backward time from *t* to 0—is a scalar field over the final positions $x_{t}$, whose high value marks the location of *attracting LCSs*, hereafter *attractors* (Fig. 2D). Therefore, given a time interval [0,t], repellers are visualized at the initial positions (marked by high values of $\Lambda_{0}^{t}(x_{0})$) while attractors at the final position (marked by high values of $\Lambda_{t}^{0}(x_{t})$). Together, $\left(\Lambda_{0}^{t},\Lambda_{t}^{0}\right)$ help identify repellers and attractors while $\left({\;}_{\mathrm{iso}}\Lambda ,{\;}_{\mathrm{aniso}}\Lambda \right)$ quantify the isotropic and anisotropic Lagrangian deformation. While there are multiple definitions and types of LCSs (19, 21, 52–56)—some of which are directly computable from $B_{0}^{t}(x_{0})$—here, we focus on the most common one—attractors and repellers based on FTLE—owing to its computational simplicity and straightforward visualization.

**Figure 2:**
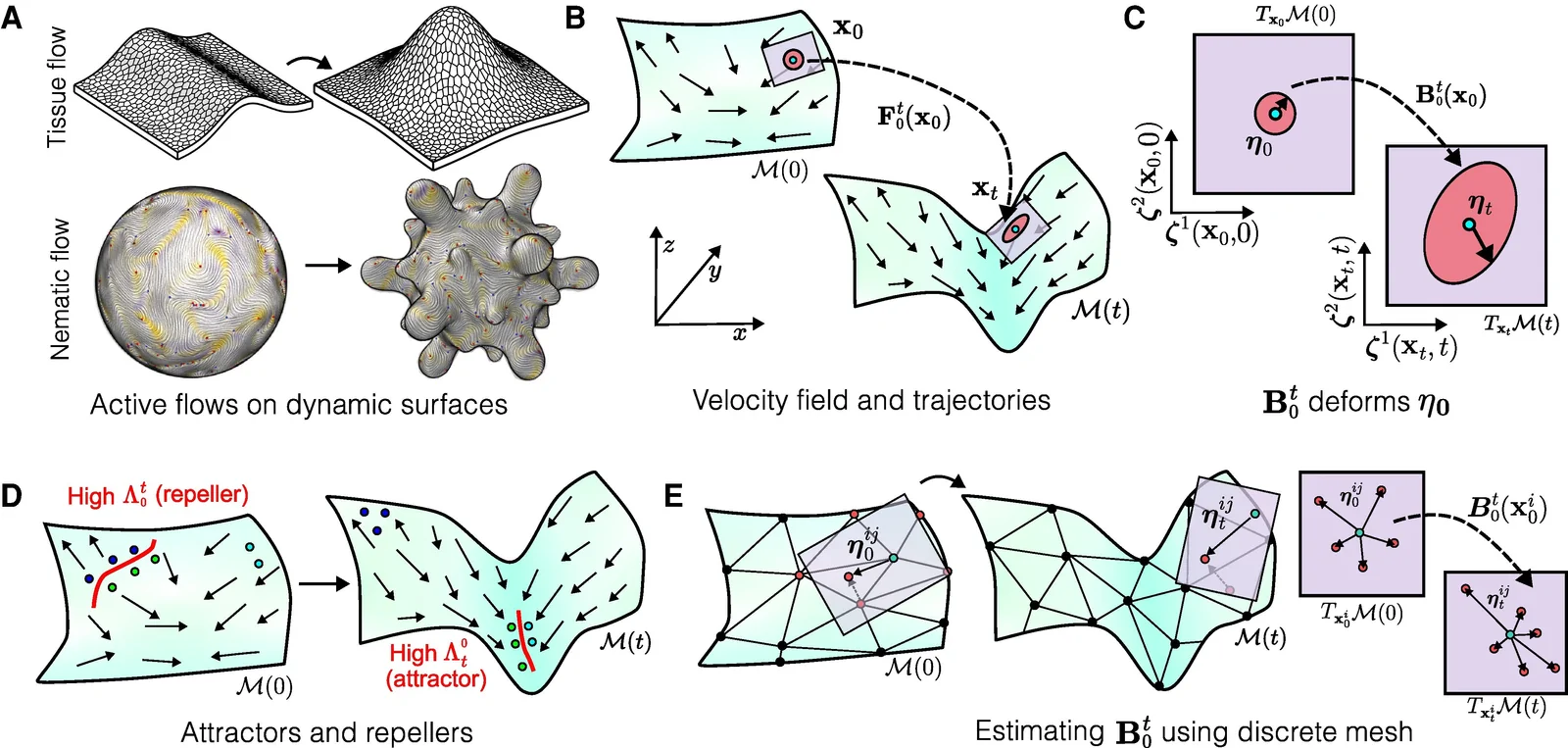
Lagrangian deformation and CSs of flows on dynamic surfaces. A) Schematic tissue and nematic active flows on dynamic surfaces. B) Flows are represented by 3D velocities v(x,t) on evolving two-dimensional surfaces M(t). The flow (or trajectory) map $F_{0}^{t}$ maps the initial position $x_{0}$ of a material particle (cyan dots) to its final position $x_{t}$. C) The material deformation experienced in the neighborhood of $x_{0}$ (pink shaded circular patch) is encoded in $B_{0}^{t}(x_{0})$ (Eq. 2), and $\left[\zeta^{1}(x,t),\zeta^{2}(x,t)]\in T_{x}M(t\right)$ represents a local orthonormal basis for the tangent space at x∈M(t) (purple square). D) Material particles (blue, green dots) on either side of a repeller—marked by high values of $\Lambda_{0}^{t}(x_{0})$—maximally separate during [0,t]. By contrast, attractors—marked by high values of $\Lambda_{t}^{0}(x_{t})$—locate final positions where initially far away particles (green, cyan dots) will maximally converge during [0,t]. E) The local deformation tensor $B_{0}^{t}(x_{0}^{i})$ is estimated by fitting $\eta_{t}^{ij}\approx B_{0}^{t}(x_{0}^{i})\eta_{0}^{ij}$ over neighboring nodes *j* whose shortest-path distance along the initial mesh edges from node *i* is less than *δ*. These neighbors are highlighted on M(0) (Section S2).

In simple cases, M(t) can be described via a parameterization x=r(p), where p denotes the parameters. In this case, the tangent space $T_{x}M(t)$ has an analytic parameterization, providing an alternative formalism to compute *Λ* and ${\;}_{\mathrm{iso}}\Lambda$ (Section S1.1). However, parameterizing dynamic surfaces and using the parameterization to quantify Lagrangian deformation induced by flows on the surface is cumbersome due to singularities of the parameterization. For example, parameterizing a two-sphere $S^{2}$ using p=(θ,ϕ) as r(p)=(sinθcosϕ,sinθsinϕ,cosθ) works everywhere except the poles (0,0,±1), where it is not invertible. For a static problem, one can add another parameterization with singularities elsewhere and switch between the two to fully parametrize $S^{2}$ (24)—i.e. construct an atlas (57). However, flows on dynamic manifolds would require a cumbersome construction of several charts (57) and sophisticated numerical schemes to switch between them while integrating trajectories.

To avoid these problems and broaden applications to experimental data, we leverage the discrete representation of manifolds as polygon meshes. Given the velocity field data on a discrete mesh (Fig. 2E), we first compute the flow map $x_{t}^{i}=F_{0}^{t}(x_{0}^{i})$ for every initial position $x_{0}^{i}\in M(0),i\in \{1,\ldots ,N\}$, where *i* indexes the mesh nodes. We construct local tangent planes and their orthonormal tangent basis vectors $\left[\zeta^{1}(x,t),\zeta^{2}(x,t)\right]$ on the mesh using the mesh connectivity (Section S2). Then, we estimate $B_{0}^{t}$ using $B_{0}^{t}(x_{0}^{i})\eta_{0}^{ij}=\eta_{t}^{ij}$, where $\eta_{0}^{ij}=[(x_{0}^{j}-x_{0}^{i}{)}^{⊤}\zeta^{1}(x_{0},0),(x_{0}^{j}-x_{0}^{i}{)}^{⊤}\zeta^{2}(x_{0},0){]}^{⊤}$ and $\eta_{t}^{ij}=[(x_{t}^{j}-x_{t}^{i}{)}^{⊤}\zeta^{1}(x_{t},t),(x_{t}^{j}-x_{t}^{i}{)}^{⊤}\zeta^{2}(x_{t},t){]}^{⊤}$ for every initially nearby node *j* within a shortest-path distance *δ* along mesh edges (Fig. 2E); see Section S2 and Fig. S2 for details. The distance *δ* sets the length scale of spatial averaging of the estimation of the deformation gradient B (Section S2.1 for details). This enables reliable estimation of the deformation gradient from noisy data on curved surfaces; see Section S2.2.2 for details. In Section S2, we assess the sensitivity of our method to average mesh size and temporal resolution (Fig. S3b), demonstrate its robustness to noise (Fig. S3d, e) and large deformations (Fig. S3f, g), and validate it against a 2D benchmark flow with known FTLE (Fig. S3h–j).

#### Eulerian CSs

LCSs organize trajectories over any time interval [0,t]. Given their time-integrative nature, however, they average over short-time variability in material transport. By contrast, Eulerian CSs (20) and instantaneous Lyapunov exponents (58) provide a rigorous framework to analyze short-time material transport—*short-time attractors* and *short-time repellers*—as well as regions and directions of distinct stretching and shrinking rates. These structures correspond to the instantaneous limit (t→0) of the corresponding LCS and are computable from the Eulerian rate of strain tensor while remaining inaccessible from Eulerian velocity snapshots (20, 25, 59) and nematic tensor (36). Specifically, while Lagrangian attractors are marked by ridges of $\Lambda_{t}^{0}(x_{t})$ and the associated contracting direction $\xi_{t}^{0}(x_{t})$, in the limit of t→0, short-time attractors are marked by trenches of $s_{1}(x,t)$ (i.e. highly negative regions of $s_{1}(x,t)$) and the direction of maximal contraction rate is marked by $e_{1}(x,t)$, where $s_{1}(x,t)\leq s_{2}(x,t)$ are the eigenvalues of the rate of strain tensor and $e_{1}(x,t),e_{2}(x,t)$ the corresponding eigenvectors (20). Similarly, short-time repellers are positive ridges of $s_{2}(x,t)$ and $e_{2}(x,t)$ marks the direction of maximal stretching rate. Here, we extend these techniques to flows on dynamic surfaces (Section S3).

LCS and Eulerian CS are complementary. For example, given a velocity dataset over $\left[0,t_{f}\right]$, computing LCSs over increasing finite time intervals $\left[0,t_{1}\right]$, $\left[0,t_{2}\right]$ etc., reveals when and where patterns of distinct Lagrangian transport and deformations emerge. By contrast, computing Eulerian CSs at different $t\in [0,t_{f}]$ reveals the spatiotemporal coordinates of instantaneous high deformation rates. Figures 3A–D, S4, and SM7 show Lagrangian attractors and repellers, and Eulerian short-time attractors and repellers, for the same active nematic system, along with typical velocity and nematic tensor plots.

**Figure 3:**
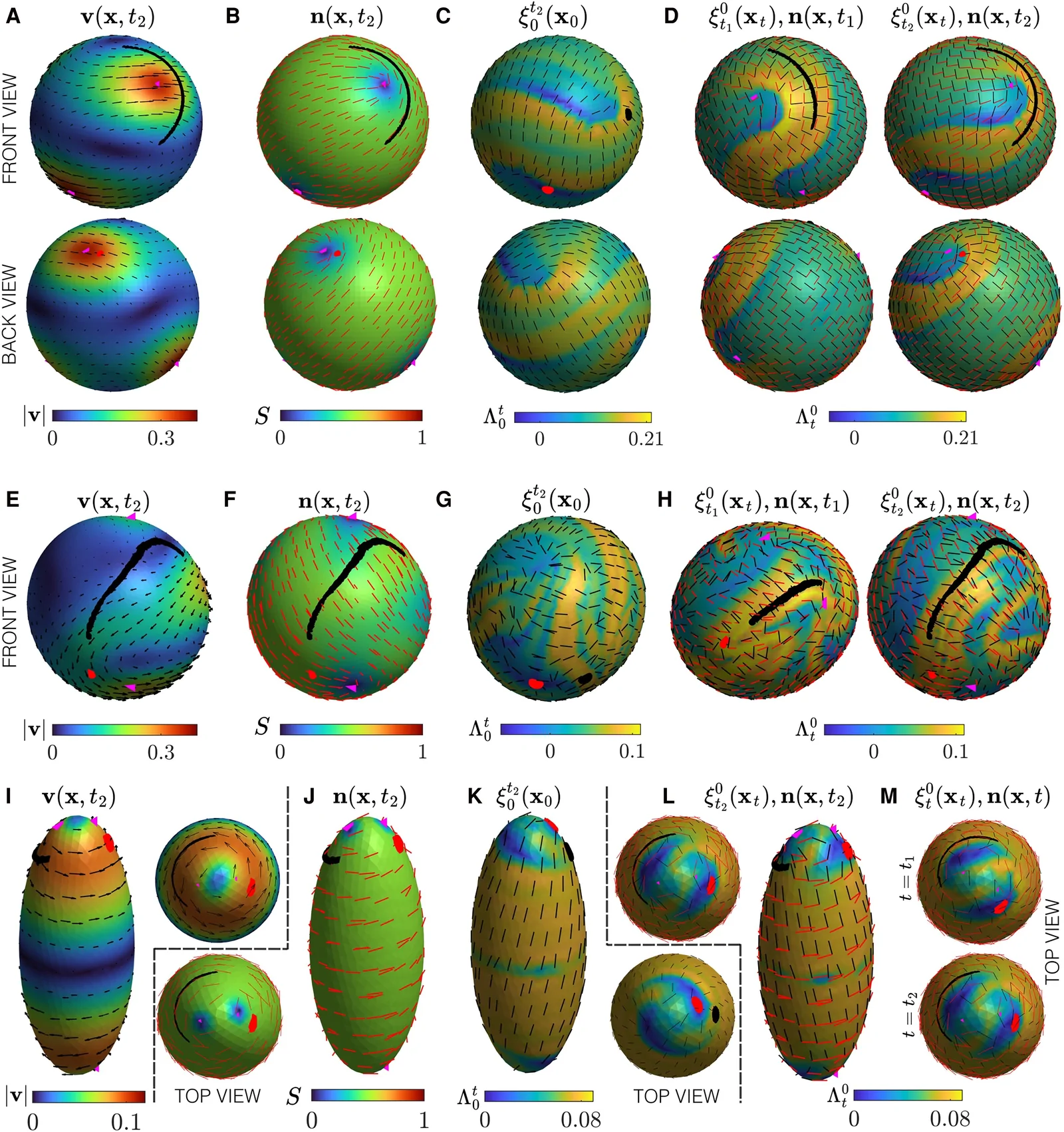
Active nematic flows on stationary and dynamic surfaces: A–D) Extensile active nematic flow on a stationary spherical surface. A) Velocity field (black arrows) $v(x,t_{2})$ with colormap denoting velocity magnitude. B) Nematic director field (red bars) $n(x,t_{2})$ and nematic order parameter $S(x,t_{2})$ (scalar field). Magenta triangles mark +1/2 topological defects. C) The forward FTLE field $\Lambda_{0}^{t_{2}}(x_{0})$ and maximum stretching (or repulsion) direction $\xi_{0}^{t_{2}}$ (black bars) for Lagrangian deformation over the time interval $\left[0,t_{2}\right]$. D) The backward FTLE field $\Lambda_{t}^{0}(x_{t})$, maximal contracting (or attracting) direction $\xi_{t}^{0}$ (black bars) and n(x,t) (red bars) for increasing *t*. A,B,D) Red and black patches mark the final position of tracer particles initialized as small disks at locations shown in (C). SM1 shows the time evolution of the Eulerian and Lagrangian quantities in a–d for different *t*. E–H) Same analysis as in a–d for an active nematic flow on a deforming vesicle. SM2 shows the time evolutions of (E–H). I–M) Same analysis as in a–d for an active nematic flow on a stationary ellipsoid geometry. SM3 shows the time evolutions of (I–M). All quantities are in nondimensional units.

### Active nematic flow on stationary and deformable surfaces

First, we consider an extensile nematic flow on a stationary surface, inspired by experiments with microtubule-kinesin motors confined on a spherical vesicle (10). While measuring in toto velocities and nematic directors in these experiments remains challenging, a recent study (60) developed a numerical scheme that recapitulates the flows observed in Ref. (10) by solving the nematic hydrodynamic equations on a spherical surface, mechanically coupled to the inner and outer fluids. These equations return the nematic tensor Q(x,t)=S(n⊗n−I/2) and the velocity v(x,t) fields, where 0<S≤1 is the nematic order parameter and n denotes the mean orientation of nematic elements. Because the nematic field is on a sphere, the net topological charge is constrained to +2, which manifests as four +1/2 defects (see Section S6 for details on how defect positions are identified). These defects are motile due to activity, and at low activity regimes, the four +1/2 defects move along the surface without annihilating each other.

At low activity, the simulations recapitulate the experimentally observed periodic motion of the four +1/2 defects from a tetrahedral to a planar configuration (10, 60), as shown in the first two columns of SM1 and Fig. 3A, B. This periodic motion provides a simple, controllable flow motif to study material transport. Figure 3C shows the $\Lambda_{0}^{t}$ field for the entire time interval $\left[0,t_{2}\right]$, revealing a band-shaped ridge (repeller) in the vesicle at its initial configuration $\left(x_{0}\right)$ where the flow will undergo maximum deformation (or separation) along the $\xi_{0}^{t_{2}}$ direction normal to the ridge. To visualize this repulsion, we initialize at time 0 tracer particles in a region with high values of $\Lambda_{0}^{t}$ (black Fig. 3C) and at low values for comparison (red Fig. 3C). Figure 3A, B shows the tracers at the final time $t_{2}$, displaying how the black patch deforms considerably compared to the red one during $\left[0,t_{2}\right]$. Owing to its cumulative Lagrangian nature, the band-shaped repeller, organizing material transport and revealing initial regions $x_{0}$ and directions of high stretching, cannot be identified from inspection of the Eulerian v and Q.

Figure 3D shows the backward FTLE $\Lambda_{t}^{0}(x_{t})$ for different *t*. High values of $\Lambda_{t}^{0}(x_{t})$ locate the dynamic position ($x_{t}$) of material patches that undergo maximal convergence during [0,t]—i.e. the attractors—along the direction $\xi_{t}^{0}$. The black patch visualizes this effect, starting initially circular and within the domain of attraction of the attractor (Section S4, Fig. S5, and SM6) and ultimately deforming and converging into the $\Lambda_{t}^{0}(x_{t})$ ridge (Fig. 3D). Similar to $\Lambda_{0}^{t}(x_{0})$ (visualized at the initial configuration), $\Lambda_{t}^{0}(x_{t})$ (visualized at the final configuration) reveals a band-shaped structure of the attractor and regions of high contraction, which are not contained in v and Q plots (Fig. 3A, B). Consistent with 2D planar active nematics (36), we find that (i) motile +1/2 defects deform the $\Lambda_{t}^{0}$ ridges and (ii) the extensile nematic director aligns to the $\Lambda_{t}^{0}$ ridge (Fig. 3D). SM1 shows the time evolution of the Eulerian and Lagrangian quantities in Fig. 3A–D. For short-time attractors and repellers associated with Fig. 3A–D, see Section S3, Fig. S4 and SM7. This low-activity vesicle system with regular +1/2 defect motion offers a simple system to engineer synthetic materials on curved surfaces. In particular, the ability of +1/2 defects to steer attractors suggests a route to controlling material transport and deformation patterns on curved surfaces via CSs. Pursuing this direction would require extending current theoretical frameworks (61) to defect control on curved surfaces, complementing the growing literature on manipulating active nematics through topological-defect steering (62, 63).

In the high activity limit, the vesicle undergoes both in-plane and out-of-plane deformations (Fig. 3E–H), as observed experimentally (10). Similar to the static nematic vesicle (Fig. 3A–D), ridges of $\Lambda_{0}^{t}$ mark regions of high Lagrangian stretching (Fig. 3G), visualized by the distinct deformation of the red and black particle patches (Fig. 3E, F, H), initialized as small circular patches at low and high $\Lambda_{0}^{t}$ (Fig. 3G). The red patch undergoes small deformation, while the black patch highly deforms and converges to the attractors marked by $\Lambda_{t}^{0}(x_{t})$ ridges along the $\xi_{t}^{0}(x_{t})$ direction (Fig. 3H). Compared to the static vesicle case, *Λ* ridges display a more complex pattern, indicating higher flow complexity. The director field n does not align with the $\Lambda_{t}^{0}$ ridges due to the higher contribution from the elastic interactions in comparison to the low activity limit. SM2 shows the time evolution of the Eulerian and Lagrangian fields associated with Fig. 3E–H. Recent advances leveraging techniques from discrete differential geometry enabled simulations of nematic-hydrodynamics flows on any curved dynamic surface (64). Our approach applies to flows on general surfaces regardless of their genus and curvature variations (Fig. 3, Fig. S6, SM8 and SM9). This enables studying the role of curvature, topology, and defect dynamics in the material transport and deformations of active nematics and multicellular flows in deforming organs and organoids. As an example, we study the nematic flows in a prolate ellipsoid, which at low activities result in the periodic motion of two +1/2 defects at the poles (Fig. 3I–M) (65). We observe that nematic flow generates a spiral-shaped $\Lambda_{0}^{t}$ ridge at the poles with a more spatially homogenous $\Lambda_{0}^{t}$ away from the poles (Fig. 3K). As before, we visualize this deformation heterogeneity by initializing tracer particles at regions of high and low $\Lambda_{0}^{t_{2}}(x_{0})$ (black and red, Fig. 3K), and show their final positions in Fig. 3I, J, along with $v(x,t_{2})$ and $Q(x,t_{2})$. Figure 3L, M shows spiral $\Lambda_{t_{2}}^{0}(x_{t_{2}})$ ridges around the poles next to +1/2 defects and Fig. 3M shows $\Lambda_{t}^{0}(x_{t})$ for increasing *t* along with the deformed material patches strongly contracting along $\xi_{t}^{0}(x_{t})$ (black patch) or not deforming where $\Lambda_{t}^{0}(x_{t})$ is low (red patch).

### Multicellular tissue flows on pancreatic spheroids

Tissue flows in epithelial sheets are ubiquitous in morphogenesis, generating the necessary motion and deformations to form a target shape (2, 66). We analyze experimental tissue flows on pancreatic organoids (6)—a spherical epithelial monolayer with an apicobasal polarity that undergoes collective cell migration. Tissue velocities are reconstructed using particle image velocimetry (PIV) from live imaging (Fig. 4A and SM4).

**Figure 4:**
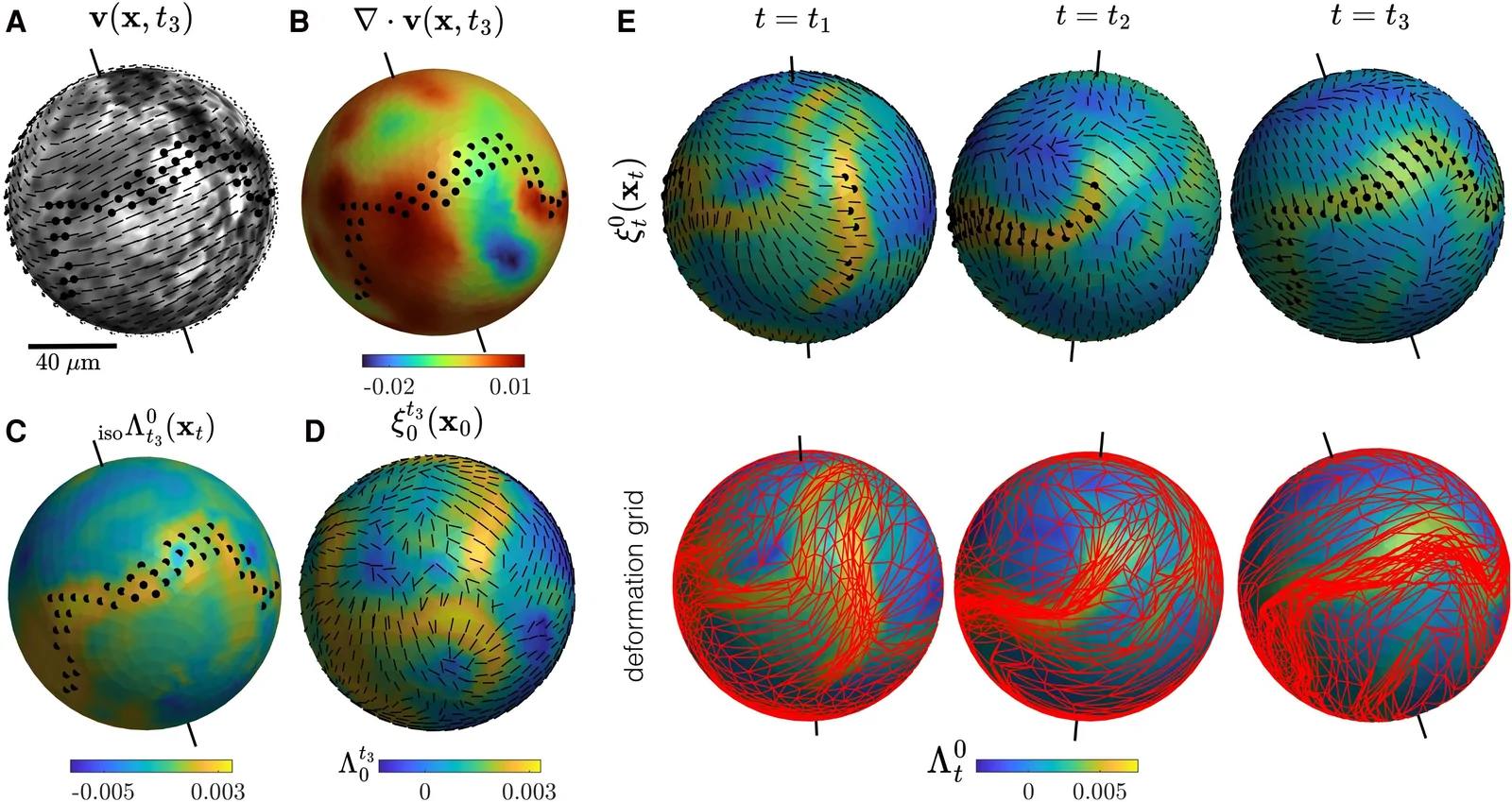
Tissue flows from collective cell migration in pancreatic spheroids: A) Velocity field overlaid on the apical surface of the spheroid at time $t_{3}=330$ min. B) Velocity field divergence $\nabla \cdot v(x,t_{3})$ associated with (A). C) Isotropic deformation field ${\;}_{\mathrm{iso}}\Lambda_{t_{3}}^{0}(x_{t})$. D) The forward FTLE $\Lambda_{0}^{t_{3}}(x_{0})$ field and the maximum stretching axis $\xi_{0}^{t_{3}}$. E) Backward FTLE $\Lambda_{t}^{0}$ for $t\in \{t_{1}=130,t_{2}=230,t_{3}=330\}$ min with the corresponding maximum shrinking direction $\xi_{t}^{0}$ (black bar) in the top row and a deforming Lagrangian grid (red) in the bottom row. A–E) The instantaneous rotational axis of the spheroid is shown in black. (See Ref. (6) for details on how the rigid rotation axis is computed from the velocity field.) Black dots in a,b,c,e mark regions of high deformation (i.e. regions with $\Lambda_{t}^{0}>$ 95th percentile at a given time). SM4 shows the time evolution of (A–E).

The flow on the surface is compressible (Fig. 4B) generating isotropic Lagrangian deformations, captured by ${\;}_{\mathrm{iso}}\Lambda_{t}^{0}(x_{t})$ (Fig. 4C). ${\;}_{\mathrm{iso}}\Lambda_{t}^{0}(x_{t})$ integrates the flow divergence experienced by a material patch along its trajectory, and high values of ${\;}_{\mathrm{iso}}\Lambda_{t}^{0}(x_{t})$ mark final-time locations of tissue patches that experienced large isotropic shrinkage. ${\;}_{\mathrm{iso}}\Lambda_{t}^{0}(x_{t})$ is the backward-time counterpart of ${\;}_{\mathrm{iso}}\Lambda_{0}^{t}(x_{0})$ (Eq. 4), whose high values mark initial positions that undergo large isotropic expansion during [0,t]. High values of $\Lambda_{0}^{t}(x_{0})$ show initial tissue regions that will undergo maximal stretching along $\xi_{0}^{t}$, which is perpendicular to the ridges (Fig. 4D). As noted before, $\Lambda_{0}^{t}(x_{0})$ ridges act as a material barrier, whereby tissue patches on either side of this ridge will maximally separate and deform. High values of $\Lambda_{t}^{0}(x_{t})$, instead, mark the final $\left(x_{t}\right)$ position of tissue regions that highly contract along $\xi_{t}^{0}$ (Fig. 4E, top). These high deformation regions are not contained in Eulerian quantities (cf. high deformation regions are marked by black dots in Fig. 4A, B, C, E and SM4). Figure 4E bottom shows a deforming Lagrangian grid and $\Lambda_{t}^{0}(x_{t})$ visualizing how grid meshes get attracted to regions of high $\Lambda_{t}^{0}(x_{t})$ along $\xi_{t}^{0}$. Overall, ${\;}_{\mathrm{iso}}\Lambda$, *Λ* and ξ fields fully characterize Lagrangian deformations of the pancreatic spheroids.

### Cyclical deformations of a zebrafish embryo heart

Large-scale tissue deformations are ubiquitous for the functioning and morphogenesis of various organs, such as the heart (17, 67), gut (68), and lungs (69). These cumulative deformations of the tissue can be quantified using Lagrangian methods. We analyze the tissue deformation in a zebrafish heartbeat at 28 hours postfertilization (hpf) reported in Ref. (12). The zebrafish heart at 28 hpf is a tubular epithelial- computable from PIV-derived tissue velocities v(x,t) (Fig. 5A, see Ref. (12) for details on velocity reconstruction). To mitigate noise in the velocity field, we set δ≈25μm, corresponding to ≈20 nearby cells for estimating the local deformation tensor. The isotropic and anisotropic Lagrangian deformations over one heartbeat period *T* are shown in Fig. 5B,C). Both return to ≈0 after one period, consistent with cardiac remodeling occurring over longer timescales. Spatial heterogeneity in deformations and their temporal dynamics could help uncover regions in the heart with differential material properties, local active stresses, as well as to investigate tissue integrity and mechanosensitive processes (17). Overall, our work provides a parameterization-free and noise-robust method to compute Lagrangian deformation and principal stretch directions from flow fields on curved, dynamic surfaces of arbitrary geometry and topology. This complements existing frameworks such as TubULAR (12), which quantifies Lagrangian deformation using parameterized tube-like surfaces. Another widely used 3D framework, MorphoGraphX (70), computes Lagrangian strain in curved, dynamic tissues from cell lineage tracking rather than from continuous flow fields. Its strain estimates, therefore, rely on persistent cell identities and are more interpretable when cell neighbor exchanges are limited.

**Figure 5:**
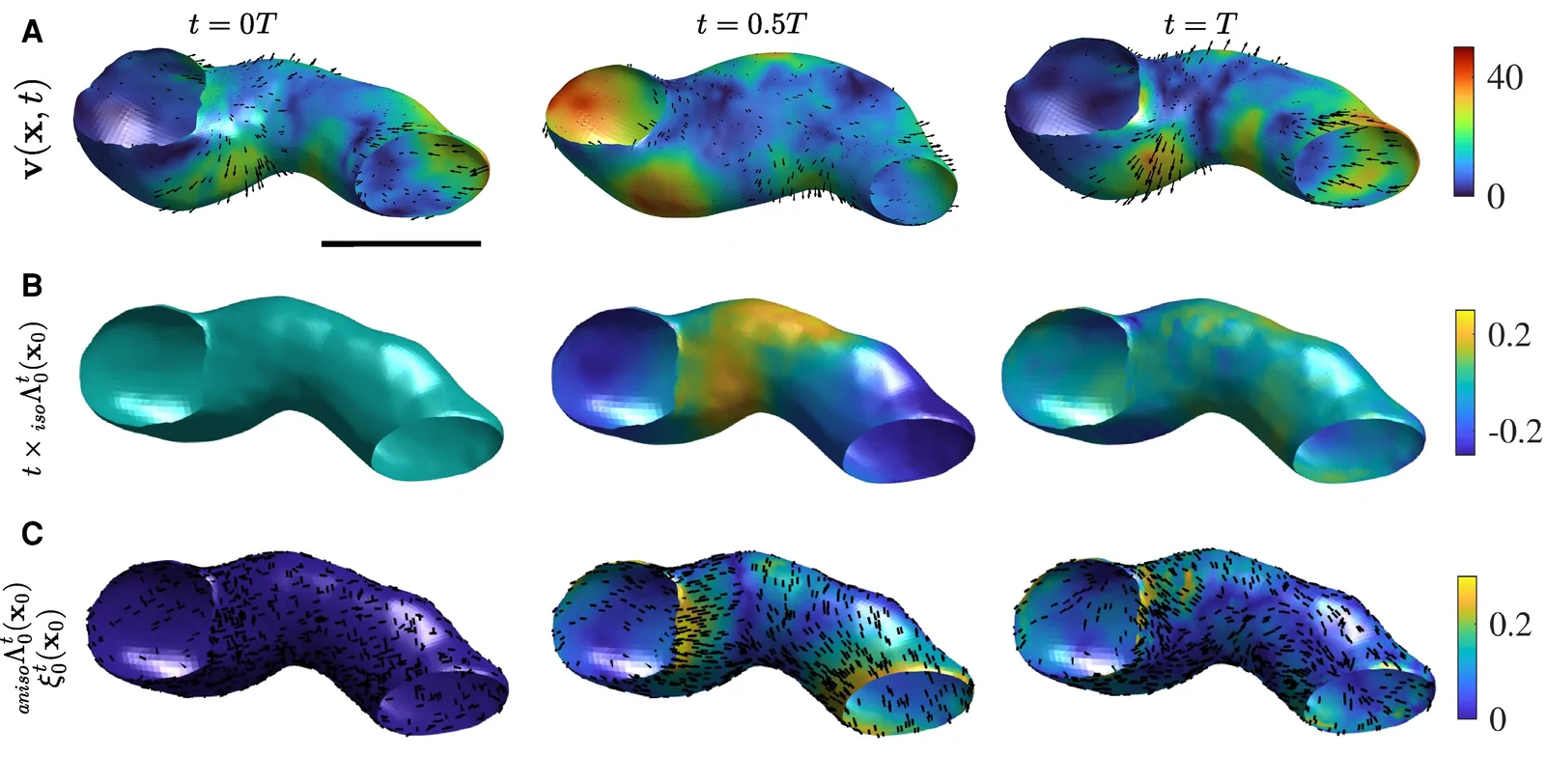
Cyclical deformations of the embryonic zebrafish heart: A–C) Quantities shown at three different times during a pumping cycle of duration *T*. A) Velocity field overlaid on the velocity magnitude in the units of μm/T. B, C) Logarithmic isotropic deformation $t\times {\;}_{\mathrm{iso}}\Lambda_{0}^{t}$ (B), deformation anisotropy ${\;}_{\mathrm{aniso}}\Lambda_{0}^{t}$ and axis of maximum deformation $\xi_{0}^{t}$ (C) visualized at the initial configuration $x_{0}$. Time evolution of (B, C) visualized at initial $x_{0}$ and final $x_{t}$ configuration is given in SM5. The scale bar is 100μm.

## Discussion

CSs organize transport and deformation in steady and unsteady flows, from turbulence and geophysical fluids to plasmas and active matter (18–21, 23–30, 33–39, 41). Yet, extending this framework to flows on dynamic, curved surfaces has remained unresolved. Here, we developed a geometric computational framework to identify LCS and Eulerian CS from velocity data on time-evolving surfaces. Our method leverages local tangent plane constructions on triangulated meshes to compute deformation tensors (Fig. 2), revealing anisotropic (${\;}_{\mathrm{aniso}}\Lambda ,\xi$) and isotropic (${\;}_{\mathrm{iso}}\Lambda$) deformation patterns and dynamic attractors and repellers on evolving manifolds. It generalizes classic LCS techniques (18, 19, 56) and recent developments in Eulerian CSs (20, 58) to dynamic surfaces with arbitrary curvature. Unlike most instantaneous flow diagnostics, CSs provide a reduced, frame-invariant description of deforming tissue flows—i.e. invariant to the arbitrary choice of reference frame used to describe motion and hence insensitive to drifts and global rotations. Furthermore, being kinematic, CSs can be computed directly from experimental velocity fields without requiring knowledge of the underlying forces.

Applying this framework to active nematic vesicles (10, 60) (Figs. 3, S4, and S5, SM6 and SM7), we uncover Lagrangian attractors and repellers whose spatial patterns and deformation directions are mediated by topological defects and surface geometry. These structures—absent from instantaneous velocity or nematic tensor fields—capture the cumulative effects of activity and geometric frustration, revealing a hidden skeleton of transport that governs how material is stretched, compressed, and segregated. Our results synergize with recent advances linking defect dynamics to flow structures and mixing (35–37), curvature-mediated defect dynamics (11, 62), and optimal control in active systems (45, 71). These connections highlight emerging opportunities to program material properties by coupling geometry and activity on dynamic surfaces.

Beyond synthetic systems, we apply our framework to collective cell motion in pancreatic spheroids (6) (Fig. 4) and the beating embryonic zebrafish heart (12) (Fig. 5). These biological tissues display dynamically evolving repellers, attractors, and anisotropic deformation axes—kinematic features that may mediate the mechanics of morphogenesis (17, 72, 73), reveal directional cues (74, 75), or regulate mechanosensitive signaling (76–78). Despite their ubiquity in living and synthetic active matter, flows on deforming surfaces have remained inaccessible to previous LCS methods, even though LCS analysis revealed new biological and physical insights when applied to multicellular flows in fixed 2D and 3D domains. For example, recent work on 2D MDCK cell cultures showed that tissue-scale LCSs mediate intercellular stress organization (42). Furthermore, tissue-scale LCSs and Lagrangian deformation axes ξ in 2D fluidized epithelia during chick gastrulation revealed barriers and enhancers of diffusing morphogen transport (see, e.g. Fig. 4 of (41)) and new mechanisms regulating the dynamics of embryo geometry (40). In addition, repellers emerging from 3D mesenchymal cell flows during zebrafish tailbud elongation preceded and biased cell-fate bifurcations (43). These examples show that tissue-scale LCSs provide useful information even in fluidized tissues. However, when the goal is to infer precise cell-scale deformation patterns and anisotropies, this interpretation is most direct when topological rearrangements are minor (79).

This framework opens avenues for future exploration. Theoretically, it enables the formulation of inverse problems, such as determining optimal activity or curvature to engineer desired material transport (45, 63, 71, 80). Experimentally, it invites integration with emerging high-resolution tissue imaging and cell tracking platforms (13–15, 43), facilitating inference of mechanical cues from motion measurements. More broadly, by enabling CS analysis on dynamic manifolds, this work provides a kinematic characterization of active and living matter, naturally integrating along trajectories local and global mechanisms across scales, with applications from contractile gels (4, 5) to developing organs (3, 12, 17, 67).

## Supplementary Material

pgag335_Supplementary_Data

## Data Availability

Sample data, code, and scripts used in this work are available at https://github.com/SreejithSanthosh/flow_coherent_structure.git. A tutorial for the code is available at https://sreejithsanthosh.github.io/FTLEhub/, and a brief tutorial is also provided in Section S5. The dataset used in Fig. 5 is publicly available through (12). The datasets used in Figs. 3 and 4 are associated with previously published studies (6, 60, 64) from groups other than ours. These collaborator-controlled datasets are not currently available in a public repository and are available from the corresponding authors of (6, 60, 64).

## Preprints

This manuscript was posted as a preprint on bioRxiv: https://www.biorxiv.org/content/10.1101/2025.05.23.655805v1.

## References

[1] Osterfield M, Du X, Schüpbach T, Wieschaus E, Shvartsman SY. 2013. Three-dimensional epithelial morphogenesis in the developing *Drosophila* egg. Dev Cell. 24:400–410. 10.1016/j.devcel.2013.01.017 PMC4080892 23449472

[2] Mitchell NP, et al 2022. Visceral organ morphogenesis via calcium-patterned muscle constrictions. Elife. 11:e77355. 10.7554/eLife.77355 PMC9275821 35593701

[3] Streichan SJ, Lefebvre MF, Noll N, Wieschaus EF, Shraiman BI. 2018. Global morphogenetic flow is accurately predicted by the spatial distribution of myosin motors. Elife. 7:e27454. 10.7554/eLife.27454 PMC5843464 29424685

[4] Turlier H, Audoly B, Prost J, Joanny J-F. 2014. Furrow constriction in animal cell cytokinesis. Biophys J. 106:114–123. 10.1016/j.bpj.2013.11.014 PMC3907238 24411243

[5] Joanny J-F, Prost J. 2009. Active gels as a description of the actin-myosin cytoskeleton. HFSP J. 3:94–104. 10.2976/1.3054712 PMC2707794 19794818

[6] Tan TH, et al 2024. Emergent chirality in active solid rotation of pancreas spheres. PRX Life. 2:033006.

[7] Hirata E, Ichikawa T, Horike S-I, Kiyokawa E. 2018. Active K-Ras induces the coherent rotation of epithelial cells: a model for collective cell invasion in vitro. Cancer Sci. 109:4045–4055. 10.1111/cas.13816 PMC6272113 30281889

[8] Chin AS, et al 2018. Epithelial cell chirality revealed by three-dimensional spontaneous rotation. Proc Natl Acad Sci U S A. 115:12188–12193. 10.1073/pnas.1805932115 PMC6275504 30429314

[9] Brandstätter T, et al 2023. Curvature induces active velocity waves in rotating spherical tissues. Nat Commun. 14:1643. 10.1038/s41467-023-37054-2 PMC10039078 36964141

[10] Keber FC, et al 2014. Topology and dynamics of active nematic vesicles. Science. 345:1135–1139. 10.1126/science.1254784 PMC4401068 25190790

[11] Ellis PW, et al 2018. Curvature-induced defect unbinding and dynamics in active nematic toroids. Nat Phys. 14:85–90.

[12] Mitchell NP, Cislo DJ. 2023. TubULAR: tracking in toto deformations of dynamic tissues via constrained maps. Nat Methods. 20:1980–1988. 10.1038/s41592-023-02081-w PMC10848277 38057529

[13] Eckert J, et al 2025. Capturing nematic order on tissue surfaces of arbitrary geometry. Nat Commun. 16:7596. 10.1038/s41467-025-62694-x PMC12356910 40817333

[14] Claussen NH, Regis C, Wopat S, Lefebvre MF, Streichan SJ. 2025. Blender tissue cartography: an intuitive tool for the analysis of dynamic 3D microscopy data, bioRxiv, preprint: not peer reviewed. 10.1242/dev.205909 42929470

[15] Denberg D, et al 2024. Computing whole embryo strain maps during gastrulation. Biophys J. 123:3911–3922. 10.1016/j.bpj.2024.10.003 PMC11617634 39385470

[16] Morishita Y, Hironaka K-I, Lee S-W, Jin T, Ohtsuka D. 2017. Reconstructing 3D deformation dynamics for curved epithelial sheet morphogenesis from positional data of sparsely-labeled cells. Nat Commun. 8:15. 10.1038/s41467-017-00023-7 PMC5432036 28465614

[17] Chan Jin Jie C, et al 2025. Mechanical fracturing of the extracellular matrix patterns the vertebrate heart, bioRxiv, preprint: not peer reviewed.

[18] Shadden SC, Lekien F, Marsden JE. 2005. Definition and properties of Lagrangian coherent structures from finite-time Lyapunov exponents in two-dimensional aperiodic flows. Physica D. 212:271–304.

[19] Haller G . 2015. Lagrangian coherent structures. Annu Rev Fluid Mech. 47:137–162.

[20] Serra M, Haller G. 2016. Objective Eulerian coherent structures. Chaos. 26:053110. 10.1063/1.4951720 27249950

[21] Froyland G, Padberg K. 2009. Almost-invariant sets and invariant manifolds—connecting probabilistic and geometric descriptions of coherent structures in flows. Physica D. 238:1507–1523.

[22] Truesdell C, Noll W. The non-linear field theories of mechanics. Springer, 2004.

[23] Peacock T, Dabiri J. 2010. Introduction to focus issue: Lagrangian coherent structures. Chaos. 20:017501. 10.1063/1.3278173 20370291

[24] Serra M, Sathe P, Beron-Vera F, Haller G. 2017. Uncovering the edge of the polar vortex. J Atmos Sci. 74:3871–3885.

[25] Serra M, et al 2020. Search and rescue at sea aided by hidden flow structures. Nat Commun. 11:2525. 10.1038/s41467-020-16281-x PMC7250873 32457536

[26] Beron-Vera FJ, Olascoaga MJ, Goni G. 2008. Oceanic mesoscale eddies as revealed by Lagrangian coherent structures. Geophys Res Lett. 35:L12603.

[27] Rypina II, et al 2007. On the Lagrangian dynamics of atmospheric zonal jets and the permeability of the stratospheric polar vortex. J Atmos Sci. 64:3595–3610.

[28] Mathur M, Haller G, Peacock T, Ruppert-Felsot JE, Swinney HL. 2007. Uncovering the Lagrangian skeleton of turbulence. Phys Rev Lett. 98:144502. 10.1103/PhysRevLett.98.144502 17501277

[29] Green MA, Rowley CW, Haller G. 2007. Detection of Lagrangian coherent structures in three-dimensional turbulence. J Fluid Mech. 572:111–120.

[30] Rempel EL, et al 2023. Lagrangian coherent structures in space plasmas. Rev Mod Plasma Phys. 7:32.

[31] Shadden SC, Taylor CA. 2008. Characterization of coherent structures in the cardiovascular system. Ann Biomed Eng. 36:1152–1162. 10.1007/s10439-008-9502-3 PMC3886852 18437573

[32] Green MA, Rowley CW, Smits AJ. 2010. Using hyperbolic Lagrangian coherent structures to investigate vortices in bioinspired fluid flows. Chaos. 20:017510. 10.1063/1.3270045 PMC2814836 20370300

[33] Peng J, Dabiri J. 2009. Transport of inertial particles by Lagrangian coherent structures: application to predator–prey interaction in jellyfish feeding. J Fluid Mech. 623:75–84.

[34] Wu Z, et al 2024. Coherent spore dispersion via drop-leaf interaction. Sci Adv. 10:eadj8092. 10.1126/sciadv.adj8092 PMC10830116 38295175

[35] Tan AJ, et al 2019. Topological chaos in active nematics. Nat Phys. 15:1033–1039.

[36] Serra M, Lemma L, Giomi L, Dogic Z, Mahadevan L. 2023. Defect-mediated dynamics of coherent structures in active nematics. Nat Phys. 19:1355–1361.

[37] Head LC, et al 2024. Spontaneous self-constraint in active nematic flows. Nat Phys. 20:492–500.

[38] Serra M, Streichan S, Chuai M, Weijer CJ, Mahadevan L. 2020. Dynamic morphoskeletons in development. Proc Natl Acad Sci U S A. 117:11444–11449. 10.1073/pnas.1908803117 PMC7260943 32381735

[39] Serra M, et al 2023. A mechanochemical model recapitulates distinct vertebrate gastrulation modes. Sci Adv. 9:eadh8152. 10.1126/sciadv.adh8152 PMC10699781 38055823

[40] Serrano Nájera G, Plum AM, Steventon B, Weijer CJ, Serra M. 2025. Control of tissue flows and embryo geometry in avian gastrulation. Nat Commun. 16:5174. 10.1038/s41467-025-60249-8 PMC12137940 40467548

[41] Plum AM, Serra M. 2025. Morphogen patterning in dynamic tissues. PRX Life. 3:043009. 10.1103/h74q-3dgj PMC13045748 41939013

[42] Ma T, Grudtsyna V, Bölsterli RV, Doostmohammadi A. 2025. Cellular flow architecture exposes the hidden mechanics of biological matter. PRX Life. 3:043012.

[43] Lange M, et al 2024. A multimodal zebrafish developmental atlas reveals the state-transition dynamics of late-vertebrate pluripotent axial progenitors. Cell. 187:6742–6759.e17. 10.1016/j.cell.2024.09.047 39454574

[44] Guillot C, Djeffal Y, Serra M, Pourquié O 2024. Control of epiblast cell fate by mechanical cues, bioRxiv 2024.06.24.600402, preprint: not peer reviewed.

[45] Sinigaglia C, Braghin F, Serra M. 2024. Optimal control of short-time attractors in active nematics. Phys Rev Lett. 132:218302. 10.1103/PhysRevLett.132.218302 38856253

[46] Mowlavi S, Serra M, Maiorino E, Mahadevan L. 2022. Detecting Lagrangian coherent structures from sparse and noisy trajectory data. J Fluid Mech. 948:A4.

[47] Harms TD, Brunton SL, McKeon BJ. 2024. Lagrangian gradient regression for the detection of coherent structures from sparse trajectory data. R Soc Open Sci. 11:240586. 10.1098/rsos.240586 PMC11529625 39493296

[48] Froyland G, Padberg-Gehle K. 2015. A rough-and-ready cluster-based approach for extracting finite-time coherent sets from sparse and incomplete trajectory data. Chaos. 25:087406. 10.1063/1.4926372 26328577

[49] Banisch R, Koltai P. 2017. Understanding the geometry of transport: diffusion maps for Lagrangian trajectory data unravel coherent sets. Chaos. 27:035804. 10.1063/1.4971788 28364763

[50] Lekien F, Ross SD. 2010. The computation of finite-time Lyapunov exponents on unstructured meshes and for non-Euclidean manifolds. Chaos. 20:017505. 10.1063/1.3278516 20370295

[51] Haller G, Yuan G. 2000. Lagrangian coherent structures and mixing in two-dimensional turbulence. Physica D. 147:352–370.

[52] Balasuriya S, Ouellette NT, Rypina II. 2018. Generalized Lagrangian coherent structures. Physica D. 372:31–51.

[53] Schlueter-Kuck KL, Dabiri JO. 2017. Coherent structure colouring: identification of coherent structures from sparse data using graph theory. J Fluid Mech. 811:468–486.

[54] Allshouse MR, Thiffeault J-L. 2012. Detecting coherent structures using braids. Physica D. 241:95–105.

[55] Budišić M, Mezić I. 2012. Geometry of the ergodic quotient reveals coherent structures in flows. Physica D. 241:1255–1269.

[56] Hadjighasem A, Farazmand M, Blazevski D, Froyland G, Haller G. 2017. A critical comparison of Lagrangian methods for coherent structure detection. Chaos. 27:053104. 10.1063/1.4982720 28576102

[57] Kühnel W . Differential geometry. Vol. 77. American Mathematical Society, 2015.

[58] Nolan PJ, Serra M, Ross SD. 2020. Finite-time Lyapunov exponents in the instantaneous limit and material transport. Nonlinear Dyn. 100:3825–3852.

[59] Kunz L, Griesel A, Eden C, Duran R, Sainte-Rose B. 2024. Transient attracting profiles in the Great Pacific Garbage Patch. Ocean Sci. 20:1611–1630.

[60] Firouznia M, Saintillan D. 2025. Self-organized dynamics of a viscous drop with interfacial nematic activity. Phys Rev Res. 7:L012054.

[61] Shankar S, Souslov A, Bowick MJ, Marchetti MC, Vitelli V. 2022. Topological active matter. Nat Rev Phys. 4:380–398.

[62] Pearce D, Ellis PW, Fernandez-Nieves A, Giomi L. 2019. Geometrical control of active turbulence in curved topographies. Phys Rev Lett. 122:168002. 10.1103/PhysRevLett.122.168002 31075037

[63] Memarian FL, et al 2024. Controlling chaos: periodic defect braiding in active nematics confined to a cardioid. Phys Rev Lett. 132:228301. 10.1103/PhysRevLett.132.228301 38877912

[64] Zhu C, Saintillan D, Chern A. 2025. Active nematic fluids on Riemannian two-manifolds. Proc R Soc A. 481:20240418.

[65] Nestler M, Voigt A. 2022. Active nematodynamics on curved surfaces – the influence of geometric forces on motion patterns of topological defects. *Commun Comput Phys*. 31:947–965. 10.4208/cicp.OA-2021-0206

[66] Collinet C, Lecuit T. 2021. Programmed and self-organized flow of information during morphogenesis. Nat Rev Mol Cell Biol. 22:245–265. 10.1038/s41580-020-00318-6 33483696

[67] Sánchez-Posada J, Derrick CJ, Noël ES, Hughes SM. 2025. *morphoHeart*: a quantitative tool for integrated 3D morphometric analyses of heart and ECM during embryonic development. PLoS Biol. 23:e3002995. 10.1371/journal.pbio.3002995 PMC11778784 39879226

[68] Shyer AE, et al 2013. Villification: how the gut gets its villi. Science. 342:212–218. 10.1126/science.1238842 PMC4045245 23989955

[69] Palmer MA, et al 2021. Stress ball morphogenesis: how the lizard builds its lung. Sci Adv. 7:eabk0161. 10.1126/sciadv.abk0161 PMC8694616 34936466

[70] Barbier de Reuille P, et al 2015. MorphoGraphX: a platform for quantifying morphogenesis in 4D. Elife. 4:e05864. 10.7554/eLife.05864 PMC4421794 25946108

[71] Norton MM, Grover P, Hagan MF, Fraden S. 2020. Optimal control of active nematics. Phys Rev Lett. 125:178005. 10.1103/PhysRevLett.125.178005 33156653

[72] Irvine KD, Shraiman BI. 2017. Mechanical control of growth: ideas, facts and challenges. Development. 144:4238–4248. 10.1242/dev.151902 PMC5769630 29183937

[73] Valet M, Siggia ED, Brivanlou AH. 2022. Mechanical regulation of early vertebrate embryogenesis. Nat Rev Mol Cell Biol. 23:169–184. 10.1038/s41580-021-00424-z 34754086

[74] Ladoux B, Mège R. 2017. Mechanobiology of collective cell behaviours. Nat Rev Mol Cell Biol. 18:743–757. 10.1038/nrm.2017.98 29115298

[75] Dye NA, et al 2021. Self-organized patterning of cell morphology via mechanosensitive feedback. Elife. 10:e57964. 10.7554/eLife.57964 PMC8133777 33769281

[76] Halder G, Dupont S, Piccolo S. 2012. Transduction of mechanical and cytoskeletal cues by YAP and TAZ. Nat Rev Mol Cell Biol. 13:591–600. 10.1038/nrm3416 22895435

[77] Alasaadi DN, Mayor R. 2024. Mechanically guided cell fate determination in early development. Cell Mol Life Sci. 81:242. 10.1007/s00018-024-05272-6 PMC11136904 38811420

[78] Chan CJ, Heisenberg C-P, Hiiragi T. 2017. Coordination of morphogenesis and cell-fate specification in development. Curr Biol. 27:R1024–R1035. 10.1016/j.cub.2017.07.010 28950087

[79] Etournay R, et al 2015. Interplay of cell dynamics and epithelial tension during morphogenesis of the *Drosophila* pupal wing. Elife. 4:e07090. 10.7554/eLife.07090 PMC4574473 26102528

[80] Nishiyama K, et al 2025. Closed-loop control of active nematic flows. *Phys Rev X*. 15:041053.

