# Supplementary material for: Coherent structures in active flows on dynamic surfaces
Source: PNAS Nexus. 2026 Sep 30;5(11):pgag335. doi: 10.1093/pnasnexus/pgag335 (PMC13741232; doi:10.1093/pnasnexus/pgag335)
Supplement: Supplementary file 1 [file pgag335_supplementary_data.zip › PNASNEXUS-PNASNEXUS-2026-01888-T-s10.pdf]

Supplementary Information for 'Coherent Structures in Active Flows on Dynamic Surfaces'

Sreejith Santhosh[1], Cuncheng Zhu[2], Blase Fencil[1], and Mattia Serra *[1]

[1]Department of Physics, University of California, San Diego, California 92093, USA
[2]Department of Mechanical Engineering, University of California, San Diego, California 92093, USA

S1 Lagrangian deformation of dynamic surfaces

S1.1 Case 1. Surfaces defined by a parameterization

Two-dimensional surfaces embedded in three dimensions can be defined by a parameterization function $\boldsymbol{r}(\boldsymbol{p}) \in \mathbb{R}^3$ where $\boldsymbol{p} = [p^1, p^2] \in \mathbb{R}^2$ are the parameters. The surface motion is then prescribed by a three-dimensional velocity field $\boldsymbol{v}(\boldsymbol{r}(\boldsymbol{p}), t)$. The dynamic surface $\mathcal{M}(t)$ is given by the time-t position of trajectories of the initial surface points, described by the flow map (Fig. S1a)

$$\tilde{\boldsymbol{F}}_0^t(\boldsymbol{p}) = \boldsymbol{r}(\boldsymbol{p}) + \int_0^t \boldsymbol{v}(\tilde{\boldsymbol{F}}_0^\tau(\boldsymbol{p}), \tau)d\tau, \tag{S1}$$

where $\boldsymbol{r}(\boldsymbol{p}) = \boldsymbol{x}_0$ parameterizes the surface at initial time $\mathcal{M}(0)$, and $\tilde{\boldsymbol{F}}_0^t(\boldsymbol{p}) = \boldsymbol{x}_t$ parameterizes $\mathcal{M}(t)$. To characterize Lagrangian deformations, we parameterize a small neighborhood of $\boldsymbol{x}_0 = \boldsymbol{r}(\boldsymbol{p})$, using an infinitesimal vector $\boldsymbol{\delta p}$ in the parameter space, which parameterizes $\boldsymbol{\eta}_0 = \boldsymbol{\nabla_p r}(\boldsymbol{p})\boldsymbol{\delta p} \in T_{\boldsymbol{x}_0}\mathcal{M}(0)$ where $\boldsymbol{\nabla_p r}(\boldsymbol{p}) \in \mathbb{R}^{3\times 2}$. At time t, $\boldsymbol{\eta}_t = \boldsymbol{\nabla_p}\tilde{\boldsymbol{F}}_0^t(\boldsymbol{p})\boldsymbol{\delta p} \in T_{\boldsymbol{x}_t}\mathcal{M}(t)$ (Fig. S1b) where $\boldsymbol{\nabla_p}\tilde{\boldsymbol{F}}_0^t(\boldsymbol{p}) \in \mathbb{R}^{3\times 2}$. The deformation of each $\boldsymbol{\eta}_0$ characterizes the local surface deformation. At each surface point labeled by $\boldsymbol{p}$, there is a distinct $\boldsymbol{\eta}_0$ that will stretch the most—except for typically isolated locations of isotropic deformations where all $\boldsymbol{\eta}_0$ stretch equally. The finite-time Lyapunov exponent (FTLE) field $\Lambda_0^t(\boldsymbol{p})$ quantifies this Lagrangian maximal deformation rate and is given by

$$\Lambda_0^t(\boldsymbol{p}) = \max_{\boldsymbol{\delta p}} \frac{1}{|t|}\log(\frac{|\boldsymbol{\nabla_p}\tilde{\boldsymbol{F}}_0^t(\boldsymbol{p})\boldsymbol{\delta p}|}{|\boldsymbol{\nabla_p r}(\boldsymbol{p})\boldsymbol{\delta p}|}), \tag{S2}$$

where $|\cdot|$ denotes vector magnitude. Similarly, the Lagrangian isotropic deformation rate of a small neighborhood of $\boldsymbol{x}_0 = \boldsymbol{r}(\boldsymbol{p})$ is given by

$${}_{iso}\Lambda_0^t(\boldsymbol{p}) = \frac{1}{|t|}\log(\frac{|\boldsymbol{A}_t|}{|\boldsymbol{A}_0|}) = \frac{1}{2|t|}\log(\frac{\det(\boldsymbol{\nabla_p}\tilde{\boldsymbol{F}}_0^t(\boldsymbol{p})^\top \boldsymbol{\nabla_p}\tilde{\boldsymbol{F}}_0^t(\boldsymbol{p}))}{\det(\boldsymbol{\nabla_p r}(\boldsymbol{p})^\top \boldsymbol{\nabla_p r}(\boldsymbol{p}))}), \tag{S3}$$

where the local area element $\boldsymbol{A}_t = \boldsymbol{\zeta}_t^1 \times \boldsymbol{\zeta}_t^2$, $\times$ denotes the cross product and $\boldsymbol{\zeta}_t^i = \boldsymbol{\nabla_p}\tilde{\boldsymbol{F}}_0^t(\boldsymbol{p})\boldsymbol{\delta p}^i$. Here, $\boldsymbol{\delta p}^1, \boldsymbol{\delta p}^2$ are two basis vectors spanning the neighborhood of $\boldsymbol{p}$ in the parameter space and $\boldsymbol{\zeta}_t^1, \boldsymbol{\zeta}_t^2$ the corresponding (typically not orthogonal) basis vectors spanning $T_{\boldsymbol{x}_t}\mathcal{M}(t)$.

S1.2 Case 2. Non-parameterized surfaces

Typically, the velocity field $\boldsymbol{v}(\boldsymbol{x}, t)$ is provided on a curved surface defined by a discrete set of points (a mesh) without explicit parameterization. Constructing a parameterization and further computing Lagrangian quantities ${}_{iso}\Lambda_0^t$ and Λ_0^t can be cumbersome due to the presence of singularities. For example, on a unit-radius two-sphere $\mathcal{S}^2$, the parameterization $\boldsymbol{r}(\theta, \phi) = [\sin\theta\cos\phi, \sin\theta\sin\phi, \cos\theta]$ generates singularities at $\theta = 0$ and $\theta = \pi$. At the poles, the parameterization is not invertible, preventing the construction of a basis for the local tangent space and leading to numerical artifacts and instabilities. To overcome this issue, we compute the Lagrangian deformation without a surface parameterization, using the flow map

$$\boldsymbol{F}_0^t(\boldsymbol{x}_0) = \boldsymbol{x}_0 + \int_0^t \boldsymbol{v}(\boldsymbol{F}_0^\tau(\boldsymbol{x}_0), \tau)d\tau, \tag{S4}$$

*Email for correspondence: mserra@ucsd.edu

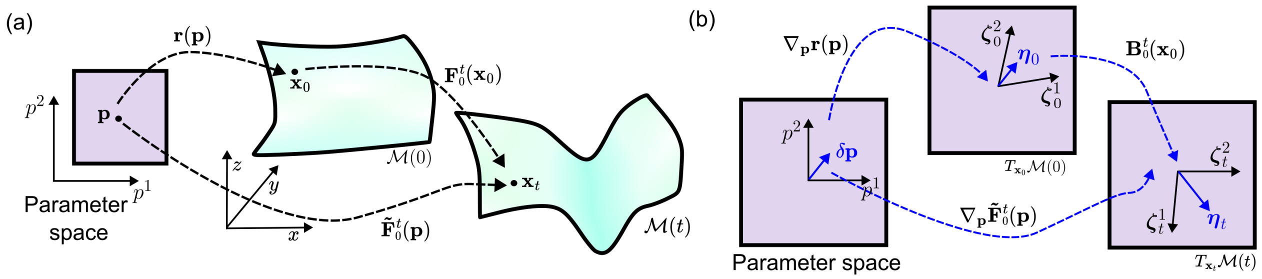

Fig. S1: **Motion and deformation of a parameterized material surface**: (a) The parameterization $\boldsymbol{x}_0 = \boldsymbol{r}(\boldsymbol{p})$ maps points $\boldsymbol{p}$ in the parameter space to the initial position $\boldsymbol{x}_0 \in \mathcal{M}(0)$. Initial positions will move to later positions according to $\boldsymbol{x}_t = \boldsymbol{F}_0^t(\boldsymbol{x}_0) = \boldsymbol{x}_0 + \int_0^t \boldsymbol{v}(\boldsymbol{F}_0^\tau(\boldsymbol{x}_0), \tau) d\tau$. Using $\boldsymbol{x}_0 = \boldsymbol{r}(\boldsymbol{p})$, one can define $\boldsymbol{F}_0^t(\boldsymbol{r}(\boldsymbol{p})) = \tilde{\boldsymbol{F}}_0^t(\boldsymbol{p})$ (Eq. S1), which parameterizes $\mathcal{M}(t)$. (b) The matrix $\boldsymbol{\nabla}_{\boldsymbol{p}}\boldsymbol{r}(\boldsymbol{p})$ maps infinitesimal vectors $\delta\boldsymbol{p}$ in the parameter space to their corresponding $\boldsymbol{\eta}_0 \in T_{\boldsymbol{x}_0}\mathcal{M}(0)$. Similarly, the matrix $\boldsymbol{\nabla}_{\boldsymbol{p}}\tilde{\boldsymbol{F}}_0^t(\boldsymbol{p})$ maps $\delta\boldsymbol{p}$ to $\boldsymbol{\eta}_t \in T_{\boldsymbol{x}_t}\mathcal{M}(t)$.

which maps the initial position $[x_0, y_0, z_0] = \boldsymbol{x}_0 \in \mathcal{M}(0)$ to its final position $\boldsymbol{x}_t = \boldsymbol{F}_0^t(\boldsymbol{x}_0) \in \mathcal{M}(t)$ (Fig. S1a). To quantify the material surface deformations, we construct an orthonormal tangent vector basis $[\boldsymbol{\zeta}^1(\boldsymbol{x}, t), \boldsymbol{\zeta}^2(\boldsymbol{x}, t)]$ (Sec. S2 for details) for $\boldsymbol{x} \in \mathcal{M}(t)$ (Fig. S2). Then, $\boldsymbol{\eta}_0 \in T_{\boldsymbol{x}_0}\mathcal{M}(0)$ and $\boldsymbol{\eta}_t \in T_{\boldsymbol{x}_t}\mathcal{M}(t)$ can be represented as

$$\begin{cases} \boldsymbol{\eta}_0 = \boldsymbol{\zeta}^1(\boldsymbol{x}_0, 0)\alpha_0^1 + \boldsymbol{\zeta}^2(\boldsymbol{x}_0, 0)\alpha_0^2 \\ \boldsymbol{\eta}_t = \boldsymbol{\zeta}^1(\boldsymbol{x}_t, t)\alpha_t^1 + \boldsymbol{\zeta}^2(\boldsymbol{x}_t, t)\alpha_t^2, \end{cases} \tag{S5}$$

where α_t^i denotes the components of $\boldsymbol{\eta}_t$ in the local tangent space basis. Additionally, the deformation tensor $\boldsymbol{\nabla}_{\boldsymbol{x}_0}\boldsymbol{F}_0^t(\boldsymbol{x}_0)$ maps $\boldsymbol{\eta}_0$ to $\boldsymbol{\eta}_t$ by $\boldsymbol{\eta}_t = \boldsymbol{\nabla}_{\boldsymbol{x}_0}\boldsymbol{F}_0^t(\boldsymbol{x}_0)\boldsymbol{\eta}_0$. Combining this equation with Eq. (S5) gives a linear map $\boldsymbol{B}_0^t(\boldsymbol{x}_0)$ between the components of $\boldsymbol{\eta}_0$ and $\boldsymbol{\eta}_t$ represented in the local tangent-space basis:

$$\begin{pmatrix} \alpha_t^1 \\ \alpha_t^2 \end{pmatrix} = \underbrace{\begin{pmatrix} \boldsymbol{\zeta}^1(\boldsymbol{x}_t, t)^\top \boldsymbol{\nabla}_{\boldsymbol{x}_0}\boldsymbol{F}_0^t(\boldsymbol{x}_0)\boldsymbol{\zeta}^1(\boldsymbol{x}_0, 0) & \boldsymbol{\zeta}^1(\boldsymbol{x}_t, t)^\top \boldsymbol{\nabla}_{\boldsymbol{x}_0}\boldsymbol{F}_0^t(\boldsymbol{x}_0)\boldsymbol{\zeta}^2(\boldsymbol{x}_0, 0) \\ \boldsymbol{\zeta}^2(\boldsymbol{x}_t, t)^\top \boldsymbol{\nabla}_{\boldsymbol{x}_0}\boldsymbol{F}_0^t(\boldsymbol{x}_0)\boldsymbol{\zeta}^1(\boldsymbol{x}_0, 0) & \boldsymbol{\zeta}^2(\boldsymbol{x}_t, t)^\top \boldsymbol{\nabla}_{\boldsymbol{x}_0}\boldsymbol{F}_0^t(\boldsymbol{x}_0)\boldsymbol{\zeta}^2(\boldsymbol{x}_0, 0) \end{pmatrix}}_{\boldsymbol{B}_0^t(\boldsymbol{x}_0)} \begin{pmatrix} \alpha_0^1 \\ \alpha_0^2 \end{pmatrix}. \tag{S6}$$

Using the definition of $\boldsymbol{B}_0^t(\boldsymbol{x}_0)$ we compute the FTLE field $\Lambda_0^t(\boldsymbol{x}_0)$ and isotropic deformation ${}_{iso}\Lambda_0^t(\boldsymbol{x}_0)$ as,

$$\begin{cases} \Lambda_0^t(\boldsymbol{x}_0) = \max\limits_{\boldsymbol{\eta}_0} \frac{1}{|t|}\log(\frac{|\boldsymbol{\eta}_t|}{|\boldsymbol{\eta}_0|}) = \frac{1}{|t|}\log(\lambda_0^t(\boldsymbol{x}_0)) \\ {}_{iso}\Lambda_0^t(\boldsymbol{x}_0) = \frac{1}{|t|}\log(\frac{|\boldsymbol{A}_t|}{|\boldsymbol{A}_0|}) = \frac{1}{|t|}\log(|\det(\boldsymbol{B}_0^t(\boldsymbol{x}_0))|), \end{cases} \tag{S7}$$

where $\lambda_0^t(\boldsymbol{x}_0)$ is the largest singular value of $\boldsymbol{B}_0^t(\boldsymbol{x}_0)$. Finally, we note that both Λ_0^t and ${}_{iso}\Lambda_0^t$ are invariant under arbitrary choices of the tangent space basis used to construct $\boldsymbol{B}_0^t(\boldsymbol{x}_0)$.

S2 Estimating B_0^t for flows described on a discrete mesh

S2.1 Numerical implementation details

To compute $\boldsymbol{B}_0^t$, we leverage the representation of curved surfaces as discrete meshes. Flows on a dynamic surface can be described by velocity vectors $\boldsymbol{v}(\boldsymbol{x}^i, t)$, where $\boldsymbol{x}^i$ is the position of node $i \in \{1, \ldots, N(t)\}$ representing $\mathcal{M}(t)$ (Fig. S2a). To determine the Lagrangian deformation during $[0, t]$, we compute the flow map by advecting the initial mesh, i.e., $\boldsymbol{x}_t^i = \boldsymbol{F}_0^t(\boldsymbol{x}_0^i)$, which maps mesh nodes $\boldsymbol{x}_0^i \in \mathcal{M}(0)$ at 0 to $\boldsymbol{x}_t^i \in \mathcal{M}(t)$. Using velocity data available at discrete time-steps δt, we perform advection using a Runge-Kutta 2nd order method in MATLAB, and project the current position of the mesh node to the surface $\mathcal{M}(t)$ at each time step. The accuracy of numerical advection depends on the quality of the mesh on which the velocity field is defined. Mesh faces with high aspect ratios reduce advection accuracy by degrading velocity interpolation within each face. In addition, the spatiotemporal resolution of the velocity data affects advection accuracy. We quantify the dependence on the mesh size δx and time step δt in Sec. S2.2. The matrix $\boldsymbol{B}_0^t(\boldsymbol{x}_0^i)$ requires constructing the tangent space basis vectors $[\boldsymbol{\zeta}^1, \boldsymbol{\zeta}^2]$ at $\boldsymbol{x}_0^i$ and $\boldsymbol{x}_t^i$. The tangent vectors are constructed by first computing the normal vector $\boldsymbol{\nu}(\boldsymbol{x}_t^i)$ using the built-in MATLAB function `vertexNormal`, and then finding two orthonormal vectors perpendicular to $\boldsymbol{\nu}(\boldsymbol{x}_t^i)$. The handedness (right or left) of the tangent vector basis need not be consistent between $\boldsymbol{x}_0^i$ and $\boldsymbol{x}_t^i$

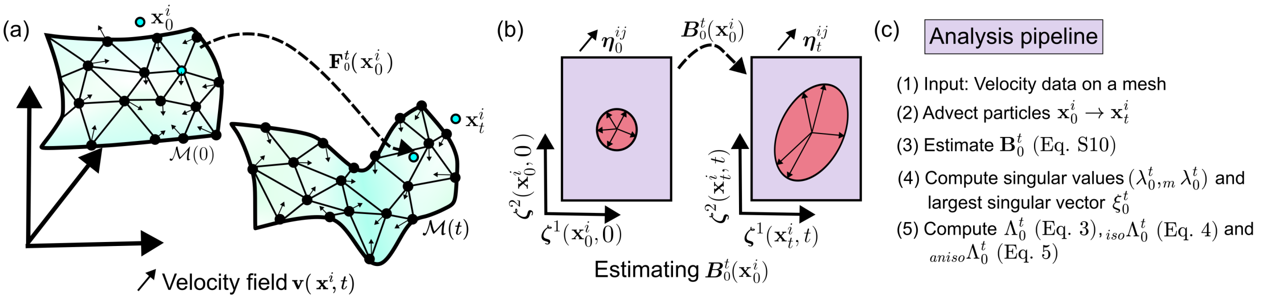

Fig. S2: **Motion and deformation of a material surface given by a discrete mesh**: (a) Flows on curved surfaces are represented by a velocity field $\boldsymbol{v}(\boldsymbol{x}^i, t)$ defined on a discrete mesh $\mathcal{M}(t)$, where $\boldsymbol{x}^i$ is a mesh node. The flow map $\boldsymbol{F}_0^t(\boldsymbol{x}_0^i)$ maps the material particle initialized at mesh node $\boldsymbol{x}_0^i$ to the final location $\boldsymbol{x}_t^i$ (typically not at a mesh node). (b) $\boldsymbol{B}_0^t(\boldsymbol{x}_0^i)$ is estimated as the linear mapping between $\boldsymbol{\eta}_0^{ij}$ and $\boldsymbol{\eta}_t^{ij}$. (c) Schematic of the numerical pipeline used to estimate $\boldsymbol{B}_0^t$, LCSs and Lagrangian deformation measures from mesh-based velocities.

as the FTLE Λ_0^t and ${}_{iso}\Lambda_0^t$ values are invariant to it.

The deformation gradient $\boldsymbol{\nabla}_{\boldsymbol{x}_0}\boldsymbol{F}_0^t(\boldsymbol{x}_0)$ quantifies the variation in the flow map with respect to variations in initial positions $\boldsymbol{x}_0$. Since the flow is restricted to a surface, the deformation gradient $\boldsymbol{\nabla}_{\boldsymbol{x}_0}\boldsymbol{F}_0^t(\boldsymbol{x}_0)$ is not defined for variation of $\boldsymbol{x}_0$ perpendicular to the material surface. Indeed, the matrix $\boldsymbol{B}_0^t(\boldsymbol{x}_0)$ (Eq. S6) restricts the variation of the deformation gradient onto the tangent space of the surface. We estimate $\boldsymbol{B}_0^t(\boldsymbol{x}_0)$ using the relation $\boldsymbol{\eta}_t = \boldsymbol{B}_0^t(\boldsymbol{x}_0)\boldsymbol{\eta}_0$ (Eqs. S5–S6) where $\boldsymbol{\eta}_0 \in T_{\boldsymbol{x}_0}\mathcal{M}(0)$ and $\boldsymbol{\eta}_t \in T_{\boldsymbol{x}_t}\mathcal{M}(t)$ (Fig. S2b). Given the flow map $\boldsymbol{F}_0^t(\boldsymbol{x}_0^i)$, we can define $(\boldsymbol{\eta}_0^{ij}, \boldsymbol{\eta}_t^{ij})$ in the local tangent vector basis as

$$\boldsymbol{\eta}_0^{ij} = \begin{pmatrix} (\boldsymbol{x}_0^j - \boldsymbol{x}_0^i)^\top \zeta^1(\boldsymbol{x}_0^i, 0) \\ (\boldsymbol{x}_0^j - \boldsymbol{x}_0^i)^\top \boldsymbol{\zeta}^2(\boldsymbol{x}_0^i, 0) \end{pmatrix}, \boldsymbol{\eta}_t^{ij} = \begin{pmatrix} (\boldsymbol{x}_t^j - \boldsymbol{x}_t^i)^\top \zeta^1(\boldsymbol{x}_t^i, t) \\ (\boldsymbol{x}_t^j - \boldsymbol{x}_t^i)^\top \boldsymbol{\zeta}^2(\boldsymbol{x}_t^i, t) \end{pmatrix}, \tag{S8}$$

where $\boldsymbol{\eta}_0^{ij}$ is the displacement from node i to node j, projected onto the local tangent space at $\boldsymbol{x}_0^i$, and $\mathcal{N}_i(\delta) = \{j \neq i : d_G(i,j) < \delta\}$ denotes the neighborhood of node i with $N_i(\delta)$ elements. Here, $d_G(i,j)$ is the shortest-path distance between nodes i and j along the mesh edges. In general, this graph distance differs from the geodesic distance on the continuous surface. The choice of δ therefore sets the spatial scale over which the local deformation tensor is estimated. Alternative neighborhoods could instead be constructed using exact geodesic distances on the triangulated surface or approximations obtained with the heat method [1]. Similarly, $\boldsymbol{\eta}_t^{ij}$ is the final position vector of the tracer particle initialized at node j relative to the final position vector of the tracer particle initialized at node i, projected onto the local tangent space at $\boldsymbol{x}_t^i$ (Fig. S2b). Because $\boldsymbol{\eta}_t^{ij} = \boldsymbol{B}_0^t(\boldsymbol{x}_0^i)\boldsymbol{\eta}_0^{ij}$ holds for every nearby node $j \in \mathcal{N}_i(\delta)$, we estimate $\boldsymbol{B}_0^t(\boldsymbol{x}_0^i)$ as

$$\boldsymbol{B}_0^t(\boldsymbol{x}_0^i) \approx \arg\min_{\boldsymbol{A}} \frac{1}{N_i}\left(\sum_j |\boldsymbol{A}\boldsymbol{\eta}_0^{ij} - \boldsymbol{\eta}_t^{ij}|^2\right), \tag{S9}$$

where $\boldsymbol{A} \in \mathbb{R}^{2\times 2}$ and $|\cdot|$ denotes the Euclidean norm. The distance δ modulates the local neighborhood size used to estimate the deformation gradient. This least-squares optimization method successfully estimated deformation gradients from sparse and noisy trajectories in 2D and 3D flows [2]. Eq. (S9) can be solved analytically and has the solution

$$\boldsymbol{B}_0^t(\boldsymbol{x}_0^i) \approx \boldsymbol{A} = (\boldsymbol{Y}\boldsymbol{X}^\top)(\boldsymbol{X}\boldsymbol{X}^\top)^{-1}, \tag{S10}$$

where the matrices $\boldsymbol{X} = (\boldsymbol{\eta}_0^{i1}, \boldsymbol{\eta}_0^{i2}, ..., \boldsymbol{\eta}_0^{iN_i(\delta)}) \in \mathbb{R}^{2\times N_i(\delta)}$ and $\boldsymbol{Y} = (\boldsymbol{\eta}_t^{i1}, \boldsymbol{\eta}_t^{i2}, ..., \boldsymbol{\eta}_t^{iN_i(\delta)}) \in \mathbb{R}^{2\times N_i(\delta)}$. In contrast to Ref. [2], we ignore the Tikhonov regularization term, as it had no significant effect in our work.

Accuracy of $\boldsymbol{B}_0^t(\boldsymbol{x}_0^i)$ estimation. To compute $\boldsymbol{B}_0^t$ using Eq. (S10), the matrix $\boldsymbol{Z}(\boldsymbol{x}_0^i) = \boldsymbol{X}\boldsymbol{X}^\top$ must be invertible. We ensure this by requiring that its condition number $\kappa(\boldsymbol{x}_0^i) \approx 1$. Large values of κ indicate that $\boldsymbol{Z}(\boldsymbol{x}_0^i)$ is ill-conditioned and may be effectively non-invertible. Since this typically occurs in regions with high-aspect-ratio initial mesh faces, we recommend remeshing these regions to make the faces more equilateral.

Furthermore, Eq. (S10) estimates $\boldsymbol{B}_0^t$ under the assumption that the deformation of $\boldsymbol{\eta}_0^{ij}$ is locally affine, as described in Eqs. (S8) and (S9). A diagnostic to check the accuracy of this approximation is the relative

residual error

$$RE(\boldsymbol{x}_0^i) = \frac{\sum_{j\in\mathcal{N}_i(\delta)} \left|\boldsymbol{B}_0^t(\boldsymbol{x}_0^i)\boldsymbol{\eta}_0^{ij} - \boldsymbol{\eta}_t^{ij}\right|}{\sum_{j\in\mathcal{N}_i(\delta)} \left|\boldsymbol{\eta}_t^{ij}\right|}. \tag{S11}$$

A large RE indicates that the local affine approximation is poor. Such errors typically occur in regions of strong deformation, where a finer mesh may be required. In addition, decreasing the neighborhood size δ can reduce RE, since a smaller neighborhood is more likely to satisfy the local affine approximation (see Secs. S2.2–S2.3 for details). Our publicly available software automatically returns warnings when κ and RE exceed allowed thresholds.

S2.2 Numerical scheme validation and sensitivity analysis

Our method for computing CSs on dynamic surfaces has two components: (i) advecting tracer particles using velocity fields on the mesh, and (ii) numerically estimating $\boldsymbol{B}_0^t$ from the initial and final tracer-particle positions and mesh connectivity. We validate the method in four ways: by quantifying advection errors as a function of spatiotemporal data resolution for an analytical three-dimensional velocity field on deforming surfaces (Fig. S3a-b); by showing that our FTLE (and CSs) identification is robust to noise (Fig. S3d-e); by studying its sensitivity to the overall deformation strength (Fig. S3f-g); and by testing our FTLE algorithm against a standard two-dimensional benchmark with known FTLE (Fig. S3h-j).

S2.2.1 Quantifying advection error

We consider the analytical velocity field given by

$$\begin{aligned} v_r &= (\theta(\theta-\pi)/5) \times e^{-(\theta-\pi/2)^2/(0.08)} \\ v_\phi &= A_\phi r \sin\theta \tanh(-5(\theta-\pi/2)) \\ v_\theta &= 0, \end{aligned} \tag{S12}$$

where (r,ϕ,θ) are the spherical coordinates and (v_r, v_ϕ, v_θ) are the components of the velocity field along the local spherical coordinate unit vectors. We generate ground-truth trajectories by integrating Eq. (S12) from a uniformly distributed set of initial conditions on a sphere of radius $r = 1$ (Fig. S3a, left) over a time interval $[0,1]$—this defines the dynamic surface. We perform integration using MATLAB's *ode45* solver with *RelTol*$= 10^{-6}$ and *AbsTol*$= 10^{-8}$. The corresponding final positions are shown in Fig. S3a, right, for the parameter value $A_\phi = 1$, which controls the overall deformation strength. To mimic a situation in which velocities are defined only on a discrete mesh, we evaluate the analytical velocity (Eq. (S12)) on uniform meshes defined on the deformed surface at intermediate times, which constitute our numerical dataset. Then, to test our advection scheme, we quantify the error distribution $e = |\boldsymbol{x}_g(t=1) - \boldsymbol{x}_m(t=1)|$ between the ground-truth final tracer-particle positions $\boldsymbol{x}_g$ and the advected positions $\boldsymbol{x}_m$, obtained from velocity data defined on meshes. Figure S3b shows that increasing the spatiotemporal resolution decreases both the mean error e and its variance, validating our advection scheme. We note that (i) errors of order 10^{-2} are negligible compared to the system size, and (ii) even decreasing δt retains a residual error due to the finite δx resolution and vice versa.

S2.2.2 Robustness of CS estimation to noise

To highlight the role of spatial averaging controlled by δ (Sec. S2.1), we compare the Λ profile computed using our approach (Eq. (S10)) with that obtained from an affine deformation gradient on each mesh face. The affine map $\boldsymbol{\nabla F}_0^t$ from the initial face $(\boldsymbol{x}_0^1, \boldsymbol{x}_0^2, \boldsymbol{x}_0^3)$ at time 0 to the final face $(\boldsymbol{x}_t^1, \boldsymbol{x}_t^2, \boldsymbol{x}_t^3)$ at time t (Fig. S3c) is

$$\boldsymbol{\nabla F}_0^t \approx (-(\boldsymbol{x}_t^2 - \boldsymbol{x}_t^1) \otimes (\boldsymbol{\nu} \times (\boldsymbol{x}_0^3 - \boldsymbol{x}_0^1)) + (\boldsymbol{x}_t^3 - \boldsymbol{x}_t^1) \otimes (\boldsymbol{\nu} \times (\boldsymbol{x}_0^2 - \boldsymbol{x}_0^1)))/(2A_0), \tag{S13}$$

where $\boldsymbol{\nu} = (\boldsymbol{x}_0^2 - \boldsymbol{x}_0^1) \times (\boldsymbol{x}_0^3 - \boldsymbol{x}_0^1)/(2A_0)$ and $A_0 = |(\boldsymbol{x}_0^2 - \boldsymbol{x}_0^1) \times (\boldsymbol{x}_0^3 - \boldsymbol{x}_0^1)|/2$ (Fig. S3c). The deformation gradient $\boldsymbol{\nabla F}_0^t$ is a 3×3 matrix. We project it onto the tangent-vector basis $(\boldsymbol{\zeta}_0^1, \boldsymbol{\zeta}_0^2)$, as described in Sec. S1.2, to compute $\boldsymbol{B}_0^t$. This provides an alternative, purely local method for computing Lagrangian deformation on each mesh face, without using neighboring faces or spatial averaging.

To illustrate the effect of spatial averaging, we use ground-truth advection tracks generated with MATLAB's *ode45* solver, as described in Sec. S2.2.1, for the velocity field in Eq. (S12) with $A_\phi = 1$, and project the particle positions onto the mesh. In the noise-free case, the Λ profiles computed using Eqs. (S10) and (S13) are similar (Fig. S3d). We then add Gaussian white noise of amplitude A_{noise} to the advected trajectories and project them back onto the mesh surface. For $A_{\text{noise}} = 0.05$, the face-wise affine method in Eq. (S13) produces a noisy Λ

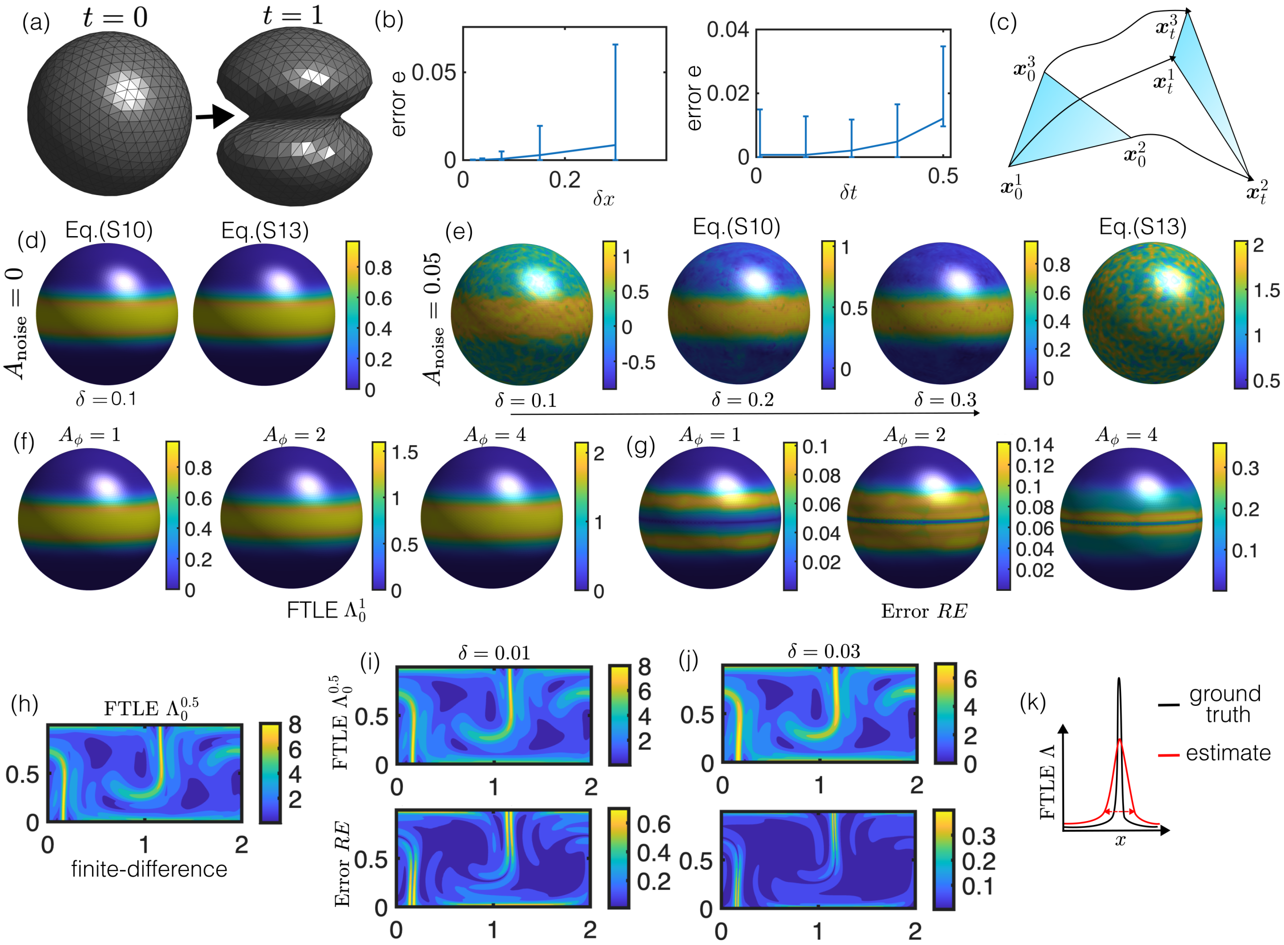

Fig. S3: **Validating our numerical method**: (a-g) Computed using the velocity field Eq. (S12). (a) Deformation of an initial sphere of radius $r = 1$ over the time interval $[0, 1]$ with $A_\phi = 1$. (b) Advection error distribution e of final tracer positions as a function of mesh size and temporal resolution $(\delta x, \delta t)$ of the velocity data. Error bars indicate the 10th and 90th percentiles. We set $\delta t = 0.01$ when varying δx, and $\delta x \approx 0.15$ when varying δt. (c) Schematic of an affine transformation mapping the mesh face $(\boldsymbol{x}_0^1, \boldsymbol{x}_0^2, \boldsymbol{x}_0^3)$ to $(\boldsymbol{x}_t^1, \boldsymbol{x}_t^2, \boldsymbol{x}_t^3)$. (d,e) Comparison of the FTLE Λ_0^1 computed using Eq. (S10) with spatial averaging over distance δ, and using Eq. (S13), with additive advection white noise: (d) $A_{\text{noise}} = 0$ and (e) $A_{\text{noise}} = 0.05$. (f,g) Λ_0^1 (f) and error RE (Eq. (S11)) (g) for varying A_ϕ. (h-j) $\Lambda_0^{0.5}$ for the double-gyre velocity arising from the stream function in Eq. (S14), computed using the finite-difference method of the flow map (h) (Sec. S2.2.4) and Eq. (S10) (i,j). In (i,j), we also visualize the error $RE(\boldsymbol{x_0})$ for varying δ. (k) Illustration of the smoothening of FTLE values across an FTLE ridge, controlled by the parameter δ.

profile, whereas the spatially averaged method in Eq. (S10) preserves the dominant deformation structure (Fig. S3e). Increasing δ further reduces noise. These results illustrate the suppression of trajectory noise by spatial averaging in Eq. (S10). Because the vertex-based and face-based estimates use different spatial neighborhoods, this example does not establish an intrinsic difference in their noise sensitivity under comparable averaging conditions.

S2.2.3 CS estimation error under large deformations

Next, we evaluate the accuracy of FTLE (and CSs) estimated from Eq. (S10) under increasing Lagrangian deformation (Fig. S3f). We evaluate the velocity field in Eq. (S12) for varying A_ϕ on meshes, as described in Sec. S2.2.1, generating datasets with different deformation strengths. We then quantify the estimation accuracy using the relative residual error RE defined in Eq. (S11). We find that RE increases with A_ϕ, particularly in regions of large deformation (Fig. S3g). Visualizing $RE(\boldsymbol{x}_0^i)$ identifies regions where the FTLE estimation is less accurate, thereby providing a diagnostic for targeted numerical refinement, such as local remeshing. In summary, the relative residual error RE provides a metric to verify the quality of our estimation. Notably, the overall shape of the LCSs identified by the FTLE field remains robust to errors in $\boldsymbol{B}_0^t$—a desirable property for noisy experimental data.

S2.2.4 Validating our algorithm against a benchmark 2D example with known FTLE

Lastly, we validate our FTLE algorithm by comparing it with the standard finite-difference computation of the flow map for a benchmark two-dimensional flow [3]. The velocity field is the double-gyre flow defined by the streamfunction

$$\Psi(x, y, t) = \sin(\pi(x - g(t))) \sin(\pi y), \tag{S14}$$

where $g(t) = 0.3 \sin(4\pi t) + 0.1 \sin(2t)$. An initial grid of points with resolution $\delta x = 0.003$ is advected over the time interval $[0, 0.5]$. The FTLE field Λ computed from finite differences of the flow map $\boldsymbol{F}_0^{0.5}(\boldsymbol{x}_0)$ is shown in Fig. S3h, while the corresponding FTLE fields computed using Eq. (S10) are shown in Fig. S3i,j. These panels confirm that our algorithm captures the correct FTLE field and LCSs, with increasingly smoothed structures as δ and δx increase. A closer inspection of Fig. S3i-j reveals that as the smoothing neighborhood size δ increases, the thickness of the estimated FTLE ridge, marking the repeller, increases. This width increase generates bands of higher RE across the repeller, without altering the correct LCS location, as illustrated in Fig. S3k. It is known that the precise estimation of peak FTLE values at repeller locations is difficult and, if desired, would require adaptive refinement [4]. LCSs, however, are concerned with the spatiotemporal structure of FTLE ridges, which remains robust even as RE increases, rather than with their precise peak values.

S2.3 Strategy for selecting the parameter δ

The distance δ sets the maximum distance between two initial nodes used to estimate the deformation gradient. We recommend starting with δ equal to the maximum mesh-edge length, δx, so that each node has at least one nearby node available for the deformation estimate. If the resulting deformation fields are noisy, or if the ridges of Λ and ${}_{iso}\Lambda$ appear at length scales comparable to the mesh size, δ should be increased.

Increasing δ reduces noise in the deformation estimates, as demonstrated in Fig. S3e and discussed in Sec. S2.2.2. However, larger values of δ also introduce stronger spatial smoothing, which can blur sharp deformation features (Fig. S3h-j). Therefore, δ should be increased only until coherent ridges emerge at length scales larger than the mesh size δx, while monitoring RE as defined in Eq. (S11). As shown in Fig. S3, our method remains robust in estimating the LCSs—namely the spatiotemporal locations of FTLE ridges—even as RE increases. This increase is mostly due to errors in the peak FTLE values at the ridges, rather than errors in their locations (Fig. S3h-k). All analyses reported in this paper use $\delta = \delta x$, unless otherwise stated.

S3 Eulerian Coherent Structures on dynamic surfaces

Recent theoretical advances [5] have established frame-invariant criteria for identifying Coherent Structures that influence material transport over short times, providing information undetectable by standard methods [6–8]. These structures include Objective Eulerian Coherent Structures [5] as well as instantaneous Lyapunov exponents [9]. They offer a mathematically rigorous instantaneous limit as $t \to 0$ for Lagrangian Coherent Structures. Eulerian Coherent Structures can be identified from the eigenvalues $s_1 \leq s_2$ and eigenvectors $[\boldsymbol{e}_1, \boldsymbol{e}_2]$ of the strain rate tensor $\boldsymbol{S} = (\boldsymbol{\nabla v} + \boldsymbol{\nabla v}^\top)/2$, quantifying instantaneous deformation rates. Here, we extend these techniques to flows on dynamic surfaces. To quantify the rate of strain tensor for flows on curved surfaces, we compute the deformation rate of $\boldsymbol{\eta} \in T_{\boldsymbol{x}}\mathcal{M}(t)$ following the setup described in Sec. S1.2, which gives

$$\frac{1}{l}\frac{dl}{dt} = \frac{\boldsymbol{\eta}^\top \boldsymbol{S} \boldsymbol{\eta}}{l^2}, \tag{S15}$$

where $l^2 = \boldsymbol{\eta}^\top \boldsymbol{\eta}$. Representing $\boldsymbol{\eta}$ in the local tangent basis $\boldsymbol{\eta} = \alpha^1 \boldsymbol{\zeta}^1(\boldsymbol{x}, t) + \alpha^2 \boldsymbol{\zeta}^2(\boldsymbol{x}, t)$, we rewrite Eq. (S15) as

$$\frac{1}{l}\frac{dl}{dt} = \frac{\boldsymbol{\alpha}^\top \boldsymbol{D} \boldsymbol{\alpha}}{\boldsymbol{\alpha}^\top \boldsymbol{\alpha}}, \quad \boldsymbol{D}(\boldsymbol{x}, t) = \begin{pmatrix} \boldsymbol{\zeta}^1(\boldsymbol{x}, t)^\top \boldsymbol{S}\, \boldsymbol{\zeta}^1(\boldsymbol{x}, t) & \boldsymbol{\zeta}^1(\boldsymbol{x}, t)^\top \boldsymbol{S}\, \boldsymbol{\zeta}^2(\boldsymbol{x}, t) \\ \boldsymbol{\zeta}^2(\boldsymbol{x}, t)^\top \boldsymbol{S}\, \boldsymbol{\zeta}^1(\boldsymbol{x}, t) & \boldsymbol{\zeta}^2(\boldsymbol{x}, t)^\top \boldsymbol{S}\, \boldsymbol{\zeta}^2(\boldsymbol{x}, t) \end{pmatrix}, \tag{S16}$$

where $\boldsymbol{\alpha} = [\alpha^1, \alpha^2]^\top$ and $\boldsymbol{D}(\boldsymbol{x}, t)$ is the rate of strain tensor projected onto the tangent plane $T_{\boldsymbol{x}}\mathcal{M}(t)$. To estimate $\boldsymbol{D}(\boldsymbol{x}^i, t)$ at mesh node $\boldsymbol{x}^i \in \mathcal{M}(t)$, following the setup described in Sec. S2, we use the relation

$$\boldsymbol{v}^{ij} = \underbrace{\begin{pmatrix} \boldsymbol{\zeta}^1(\boldsymbol{x}, t)^\top \boldsymbol{\nabla v} \boldsymbol{\zeta}^1(\boldsymbol{x}, t) & \boldsymbol{\zeta}^1(\boldsymbol{x}, t)^\top \boldsymbol{\nabla v} \boldsymbol{\zeta}^2(\boldsymbol{x}, t) \\ \boldsymbol{\zeta}^2(\boldsymbol{x}, t)^\top \boldsymbol{\nabla v} \boldsymbol{\zeta}^1(\boldsymbol{x}, t) & \boldsymbol{\zeta}^2(\boldsymbol{x}, t)^\top \boldsymbol{\nabla v} \boldsymbol{\zeta}^2(\boldsymbol{x}, t) \end{pmatrix}}_{\boldsymbol{E}(\boldsymbol{x}^i, t)} \boldsymbol{\eta}^{ij}, \tag{S17}$$

where $\boldsymbol{v}^{ij} = [(\boldsymbol{v}(\boldsymbol{x}^j, t) - \boldsymbol{v}(\boldsymbol{x}^i, t))^\top \boldsymbol{\zeta}^1(\boldsymbol{x}, t), (\boldsymbol{v}(\boldsymbol{x}^j, t) - \boldsymbol{v}(\boldsymbol{x}^i, t))^\top \boldsymbol{\zeta}^2(\boldsymbol{x}, t)]^\top$, $\boldsymbol{\eta}^{ij} = [(\boldsymbol{x}^j - \boldsymbol{x}^i)^\top \boldsymbol{\zeta}^1(\boldsymbol{x}, t), (\boldsymbol{x}^j - \boldsymbol{x}^i)^\top \boldsymbol{\zeta}^2(\boldsymbol{x}, t)]^\top$, $\boldsymbol{D}(\boldsymbol{x}^i, t) = (\boldsymbol{E}(\boldsymbol{x}^i, t) + \boldsymbol{E}(\boldsymbol{x}^i, t)^\top)/2$ and nodes i and j are neighboring nodes with shortest-path

distance less than δ. Since Eq. (S17) holds for every neighboring node $j \in \mathcal{N}_i(\delta)$, we estimate $\boldsymbol{E}(\boldsymbol{x}^i, t)$ as

$$\boldsymbol{E}(\boldsymbol{x}^i, t) \approx \arg\min_{\boldsymbol{A}} \frac{1}{N_i}\Big(\sum_j |\boldsymbol{A}\boldsymbol{\eta}^{ij} - \boldsymbol{v}^{ij}|^2\Big). \tag{S18}$$

The solution for the optimization given in Eq. (S18) is given by

$$\boldsymbol{E}(\boldsymbol{x}^i, t) \approx \boldsymbol{A} = \boldsymbol{Y}\boldsymbol{X}^\top(\boldsymbol{X}\boldsymbol{X}^\top)^{-1}, \tag{S19}$$

where $\boldsymbol{X} = (\boldsymbol{\eta}^{i1}, \boldsymbol{\eta}^{i2}, ..., \boldsymbol{\eta}^{iN_i(\delta)}) \in \mathbb{R}^{2\times N_i(\delta)}$, $\boldsymbol{Y} = (\boldsymbol{v}^{i1}, \boldsymbol{v}^{i2}, ..., \boldsymbol{v}^{iN_i(\delta)}) \in \mathbb{R}^{2\times N_i(\delta)}$ and δ sets the length scale over which the strain rate is computed. Using Eq. (S19), we compute $\boldsymbol{E}(\boldsymbol{x}^i, t)$ and its symmetric part $\boldsymbol{D}(\boldsymbol{x}^i, t)$ whose eigenvalues and corresponding eigenvectors are denoted by ($s_1 \leq s_2$) and $[\boldsymbol{e}_1, \boldsymbol{e}_2]$. Regions with high positive values of $s_2(\boldsymbol{x}, t)$ correspond to short-time repellers, and $\boldsymbol{e}_2$ marks the direction of repulsion [5]. Similarly, regions of highly negative values of $s_1(\boldsymbol{x}, t)$ mark short-time attractors and $\boldsymbol{e}_1$ the direction of attraction.

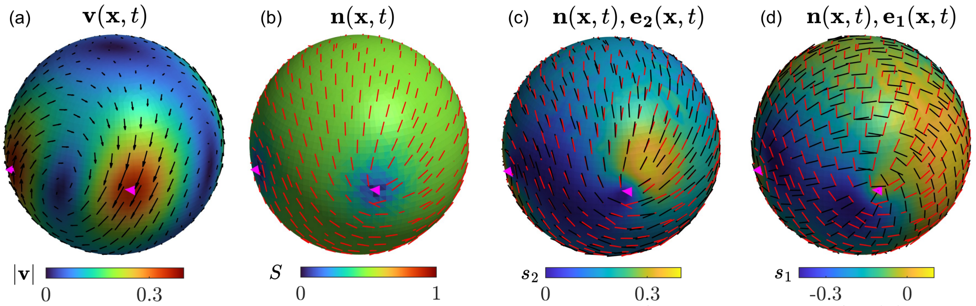

Fig. S4: **Eulerian Coherent Structures for Flows on a Vesicle (as in Fig. 3a-d)**: (a) Velocity field with the colormap denoting the velocity magnitude. (b) The nematic director field and the order parameter field. (c) The largest eigenvalue s_2 of the strain rate tensor and corresponding eigenvector $\boldsymbol{e}_2$ (black bars) along with the nematic director field (red bars). High values of s_2 mark short-time repellers or regions with the largest stretching rate. (d) The smallest eigenvalue s_1 of the strain rate tensor and the corresponding eigenvector $\boldsymbol{e}_1$, along with the nematic director field. Highly negative values of s_1 mark short-time attractors or regions with the largest shrinking rate. SM7 shows the time evolution of these quantities.

As a representative example, we compute short-time attractors and repellers (Fig. S4c-d) for the nematic flow on a vesicle analyzed in Fig. 3a-d. Fig. S4c shows a region with the highest stretching rate (high $s_2(\boldsymbol{x}, t)$) behind a $+1/2$ defect (magenta triangle), along $\boldsymbol{e}_2(\boldsymbol{x}, t)$ (black bars), aligned with the nematic director (red bars). Similarly, Fig. S4d shows a region with the highest shrinking rate (highly negative $s_1(\boldsymbol{x}, t)$) ahead of a $+1/2$ defect, along $\boldsymbol{e}_1(\boldsymbol{x}, t)$ (black bars), perpendicular to the nematic director. Notably, even Eulerian Coherent Structures remain undetected by inspection of the Eulerian velocity (Fig. S4a) and nematic tensor (Fig. S4b), consistent with earlier observations [5, 10].

S4 Domain of attraction of an attractor

To identify the attracting LCSs (or attractors) in the flow over a time interval $[0, t]$, we compute the backward FTLE field $\Lambda_t^0(\boldsymbol{x}_t)$ defined on the final positions $\boldsymbol{x}_t$. Regions with a high value of Λ_t^0 mark the attractors. In general, there are multiple attractors, each with its corresponding domain of attraction (DOA) at the initial fluid configuration $\boldsymbol{x}_0$ [11]. The DOA consists of the initial regions ($\boldsymbol{x}_0$) that will move to the attractor by time t, and can be computed by visualizing $\Lambda_t^0(\boldsymbol{F}_t^0(\boldsymbol{x}_t)) = \Lambda_t^0(\boldsymbol{x}_0)$ (Fig. S5a). As an example, we visualize the DOA (Fig. S5b) for the flow on an extensile nematic vesicle in the low activity limit, analyzed in Fig. 3a-d. We observe that for each attractor band, there is a corresponding domain of attraction. Material patches that start in the DOA (violet and blue patches, Fig. S5b) deform and move to the corresponding attractors (Fig. S5c).

S5 Guide to the Accompanying Code

The code for the numerical method described in Sec. S2.1 is available in the GitHub repository `https://github.com/SreejithSanthosh/flow_coherent_structure.git` and sketched in Fig. S2c. An expanded online tutorial, together with the latest updates, is available at `https://sreejithsanthosh.github.io/FTLEhub/`. Below, we provide a brief tutorial on how to use the code.

In the repository, the folder `ftle_on_curved_surface` contains the code for computing coherent structures

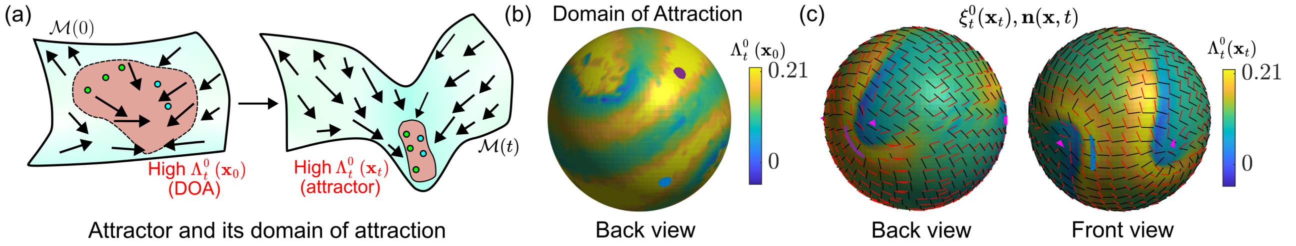

Fig. S5: **Domain of attraction (DOA)**: (a) The region with high values of the backward FTLE visualized at the initial configuration, $\Lambda_t^0(\boldsymbol{x}_0)$, marks the DOA of the corresponding attractor marked by high values of $\Lambda_t^0(\boldsymbol{x}_t)$ in the final configuration. (b) The DOA visualized using $\Lambda_t^0(\boldsymbol{x}_0)$ for flow on a static nematic vesicle analyzed in Fig. 3a-d. (c) The backward FTLE $\Lambda_t^0(\boldsymbol{x}_t)$, the largest shrinking axis $\boldsymbol{\xi}_t^0(\boldsymbol{x}_t)$ (black bars) and the nematic director field $\boldsymbol{n}(\boldsymbol{x}, t)$ (red bars). (b-c) The violet and blue patches visualize transport starting from different DOAs. The time evolution of the Eulerian and Lagrangian quantities of the flow shown in (b-c) is given in SM6.

in flows on curved surfaces. The file `example_code_lagrangian.m` provides an example MATLAB pipeline for Lagrangian coherent structure analysis. This code requires the velocity dataset to be formatted using the following variables:

1. `mesh_time`: A time vector of size $(1, N_t)$, where N_t is the number of time steps.
2. `mesh_r`: A cell array of size $(N_t, 3)$. For each time index $i \in \{1, \ldots, N_t\}$, the entries `mesh_r{i,1}`, `mesh_r{i,2}`, and `mesh_r{i,3}` are column vectors of size $(N_q(i), 1)$ containing the x-, y-, and z-coordinates of the mesh nodes, respectively. Here, $N_q(i)$ denotes the number of mesh nodes at time index i.
3. `mesh_F`: A cell array of size $(N_t, 1)$. Each entry `mesh_F{i}` gives the mesh connectivity as a matrix of size $(N_f(i), 3)$, where $N_f(i)$ is the number of mesh faces at time index i.
4. `mesh_v`: A cell array of size $(N_t, 3)$. For each time index $i \in \{1, \ldots, N_t\}$, the entries `mesh_v{i,1}`, `mesh_v{i,2}`, and `mesh_v{i,3}` are column vectors of size $(N_q(i), 1)$ containing the x-, y-, and z-components of the velocity at the mesh nodes, respectively.

A sample dataset, `example_data.mat`, is provided for reference. To run the Lagrangian analysis on a new dataset, the user should load the dataset in `example_code_lagrangian.m` by modifying the line `load('path_to_dataset')`. The user must specify the parameter δ (see Secs. S2.1 and S2.3), which sets the neighborhood distance over which deformation is estimated, and the indices `ct0` and `ctf`, which define the time interval of interest from `mesh_time(ct0)` to `mesh_time(ctf)`. Similarly, the MATLAB script `example_code_eulerian.m` provides a pipeline for Eulerian coherent structure analysis. The user should load the dataset and specify δ as above. The parameter `ct0` selects the time step at which the Eulerian analysis is performed.

S6 Identification of topological defects in active nematic flows

The flow analyzed in Fig. 3 and Fig. S6 are derived from simulations of a nematic vesicle [12] and active nematics on dynamic curved surfaces [13]. These simulations solve for the nematic tensor $\boldsymbol{Q}(\boldsymbol{x}, t) = S(\boldsymbol{n} \otimes \boldsymbol{n} - \boldsymbol{I}/2)$ and the velocity field $\boldsymbol{v}(\boldsymbol{x}, t)$, where $0 < S \leq 1$ is the nematic order parameter and $\boldsymbol{n}$ is the mean nematic orientation. For the simulations in [12], defects are identified by locating minima of S. The topological charge, computed from the director field $\boldsymbol{n}$, is then used to classify the defect type; see Supplemental Material Sec. IIIB of [12]. For the dataset in [13], defects are identified by computing the topological charge of each mesh face from the director field $\boldsymbol{n}$ defined on each vertex. Faces with nonzero charge contain a defect, and the sign and magnitude of the net charge determine its type; see Sec. 3e of [13].

S7 Additional Supplementary Figures

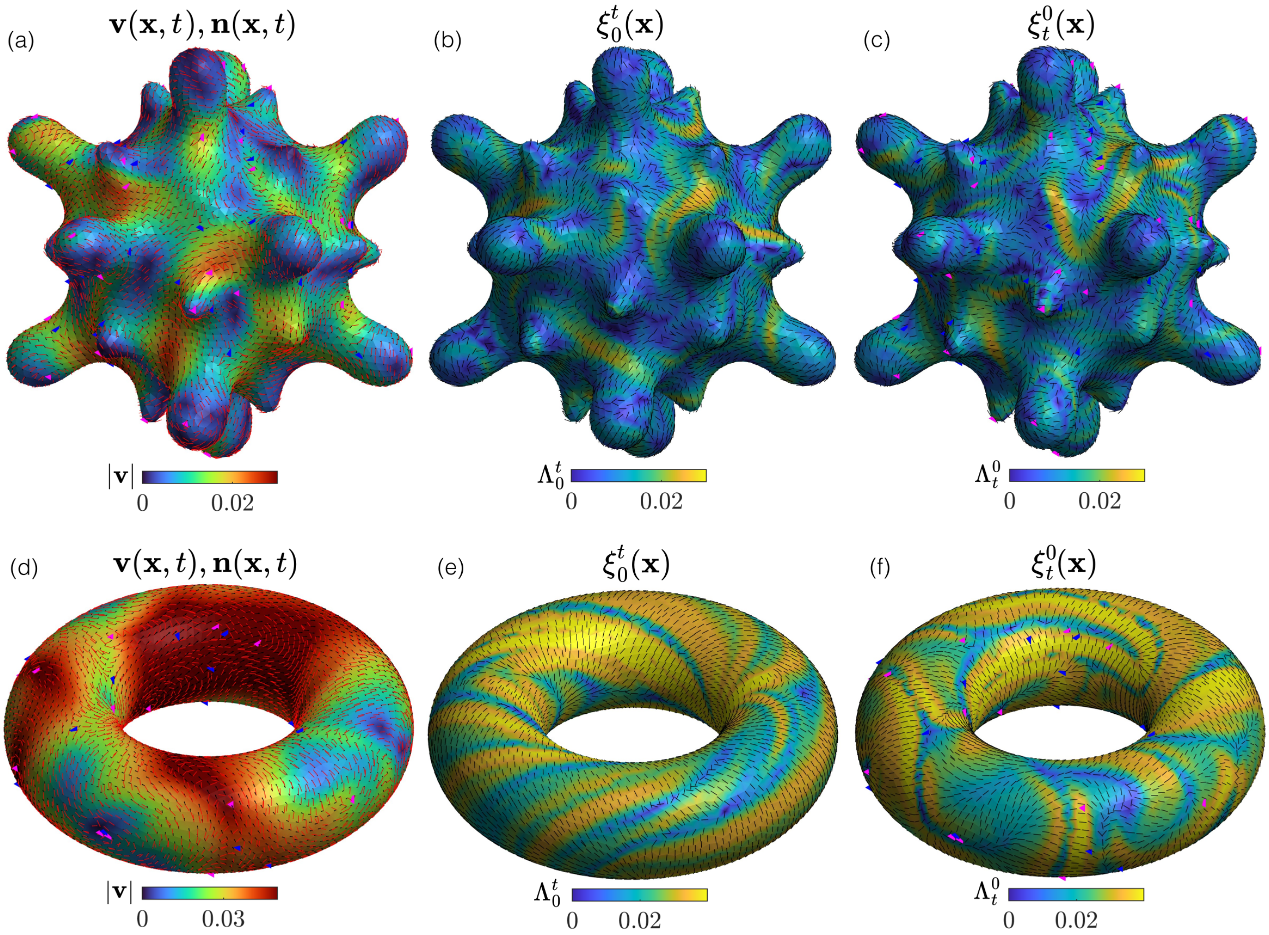

Fig. S6: **Nematic flow on a topological sphere with heterogeneous curvature and a torus.** Nematic flows are simulated using the method described in [13]. Magenta and blue triangles mark the $+1/2$ and $-1/2$ topological defects. (a) The velocity field $\boldsymbol{v}(\boldsymbol{x},t)$ (black), nematic director field $\boldsymbol{n}(\boldsymbol{x},t)$ (red) and the velocity magnitude. (b) The Λ_0^t field over the time interval $[0,t]$ and the maximum stretching axis $\boldsymbol{\xi}_0^t$. (c) The Λ_t^0 field over the time interval $[0,t]$ and maximum shrinking axis $\boldsymbol{\xi}_t^0$. SM8 shows the time evolution of the velocity field $\boldsymbol{v}(\boldsymbol{x},t)$, Λ_0^t, $\boldsymbol{\xi}_0^t$, Λ_t^0, $\boldsymbol{\xi}_t^0$ and the nematic director field $\boldsymbol{n}(\boldsymbol{x},t)$ for varying values of t. (d-f) Same as (a-c) but for nematic flow on a torus. SM9 is the same as SM8 but for the surface geometry shown in (d-f).

S8 Movie Legends

- SM1: Time evolution associated with Fig. 3a-d.
- SM2: Time evolution associated with Fig. 3e-h.
- SM3: Time evolution associated with Fig. 3i-m.
- SM4: Time evolution associated with Fig. 4.
- SM5: Time evolution associated with Fig. 5b-c.
- SM6: Time evolution associated with Fig. S5.
- SM7: Time evolution associated with Fig. S4.
- SM8: Time evolution associated with Fig. S6a-c.
- SM9: Time evolution associated with Fig. S6d-f.

References

[1] K. Crane, M. Livesu, E. Puppo, and Y. Qin, "A survey of algorithms for geodesic paths and distances," *arXiv preprint arXiv:2007.10430*, 2020.

[2] S. Mowlavi, M. Serra, E. Maiorino, and L. Mahadevan, "Detecting Lagrangian coherent structures from sparse and noisy trajectory data," *Journal of Fluid Mechanics*, vol. 948, p. A4, 2022.

[3] S. C. Shadden, F. Lekien, and J. E. Marsden, "Definition and properties of Lagrangian coherent structures from finite-time Lyapunov exponents in two-dimensional aperiodic flows," *Physica D: Nonlinear Phenomena*, vol. 212, no. 3-4, pp. 271–304, 2005.

[4] S. S. Barakat and X. Tricoche, "Adaptive refinement of the flow map using sparse samples," *IEEE Transactions on Visualization and Computer Graphics*, vol. 19, no. 12, pp. 2753–2762, 2013.

[5] M. Serra and G. Haller, "Objective Eulerian coherent structures," *Chaos: An Interdisciplinary Journal of Nonlinear Science*, vol. 26, no. 5, 2016.

[6] M. Serra, P. Sathe, I. Rypina, A. Kirincich, S. D. Ross, P. Lermusiaux, A. Allen, T. Peacock, and G. Haller, "Search and rescue at sea aided by hidden flow structures," *Nature Communications*, vol. 11, no. 1, p. 2525, 2020.

[7] R. Duran, T. Nordam, M. Serra, and C. H. Barker, "Horizontal transport in oil-spill modeling," in *Marine hydrocarbon spill assessments*, pp. 59–96, Elsevier, 2021.

[8] L. Kunz, A. Griesel, C. Eden, R. Duran, and B. Sainte-Rose, "Transient attracting profiles in the Great Pacific Garbage Patch," *Ocean Science*, vol. 20, no. 6, pp. 1611–1630, 2024.

[9] P. J. Nolan, M. Serra, and S. D. Ross, "Finite-time Lyapunov exponents in the instantaneous limit and material transport," *Nonlinear Dynamics*, vol. 100, no. 4, pp. 3825–3852, 2020.

[10] M. Serra, L. Lemma, L. Giomi, Z. Dogic, and L. Mahadevan, "Defect-mediated dynamics of coherent structures in active nematics," *Nature Physics*, vol. 19, pp. 1355–1361, 2023.

[11] M. Serra, S. Streichan, M. Chuai, C. J. Weijer, and L. Mahadevan, "Dynamic morphoskeletons in development," *Proceedings of the National Academy of Sciences*, vol. 117, no. 21, pp. 11444–11449, 2020.

[12] M. Firouznia and D. Saintillan, "Self-organized dynamics of a viscous drop with interfacial nematic activity," *Physical Review Research*, vol. 7, no. 1, p. L012054, 2025.

[13] C. Zhu, D. Saintillan, and A. Chern, "Active nematic fluids on Riemannian two-manifolds," *Proceedings of the Royal Society A*, vol. 481, no. 2311, p. 20240418, 2025.
